# Supplementary material for: Interactions of a Bacterial RND Transporter with a Transmembrane Small Protein in a Lipid Environment
Source: Structure. 2020 Jun 2;28(6):625–634.e6. doi: 10.1016/j.str.2020.03.013 (PMC7267776; doi:10.1016/j.str.2020.03.013)
Supplement: Document S1. Figures S1–S7 and Tables S1 and S3 [file mmc1.pdf]

Structure, Volume *28*

Supplemental Information

Interactions of a Bacterial RND Transporter with a Transmembrane Small Protein in a Lipid Environment

Dijun Du, Arthur Neuberger, Mona Wu Orr, Catherine E. Newman, Pin-Chia Hsu, Firdaus Samsudin, Andrzej Szewczak-Harris, Leana M. Ramos, Mekdes Debela, Syma Khalid, Gisela Storz, and Ben F. Luisi

Supplementary Information

Interactions of a bacterial RND transporter with a transmembrane small protein in a lipid environment

Dijun Du, Arthur Neuberger, Mona Wu Orr, Catherine E. Newman, Pin-Chia Hsu, Firdaus Samsudin, Andrzej Szewczak-Harris, Leana M. Ramos, Mekdes Debela, Syma Khalid, Gisela Storz, Ben F. Luisi

correspondence bfl20@cam.ac.uk, S.Khalid@soton.ac.uk, dudj@shanghaitech.edu.cn, or storzg@mail.nih.gov

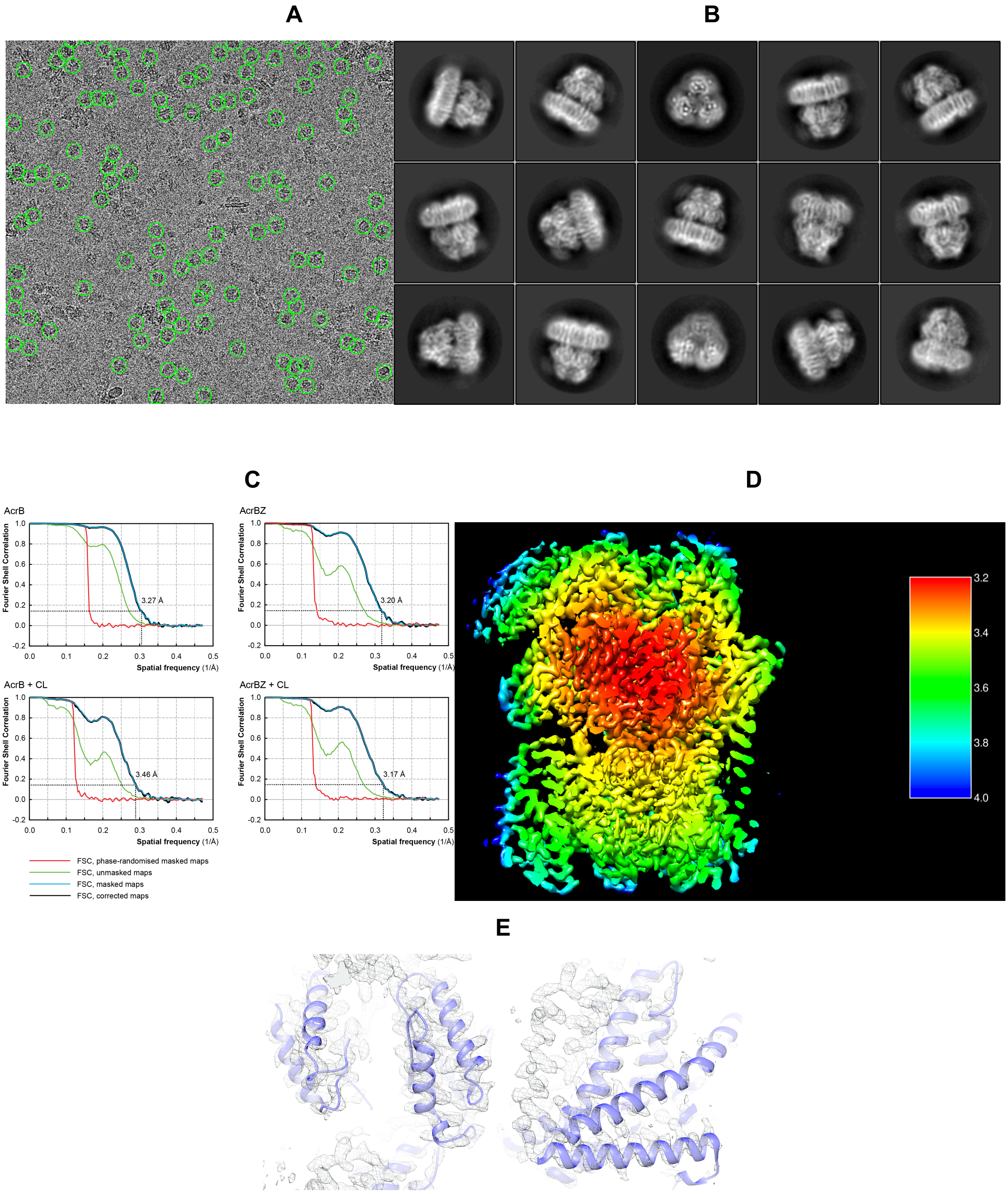

Figure S1. Related to Figure 1. Cryo-EM of AcrBZ in saposin A disc. (**A**) Representative cryo-EM micrograph image of AcrBZ in saposin A disc. The image has been corrected for drift. (**B**) Typical two-dimensional class averages of the particles. (**C**) Gold-standard Fourier Shell Correlation indicating the resolution of the density maps. Shown are FSC plots generated between reconstructions from random halves of the data. (**D**) Local resolution estimation by ResMap of AcrBZ. (**E**) Details of

structure-map overlay reveals quality of fit suitable to validate observed structural differences.

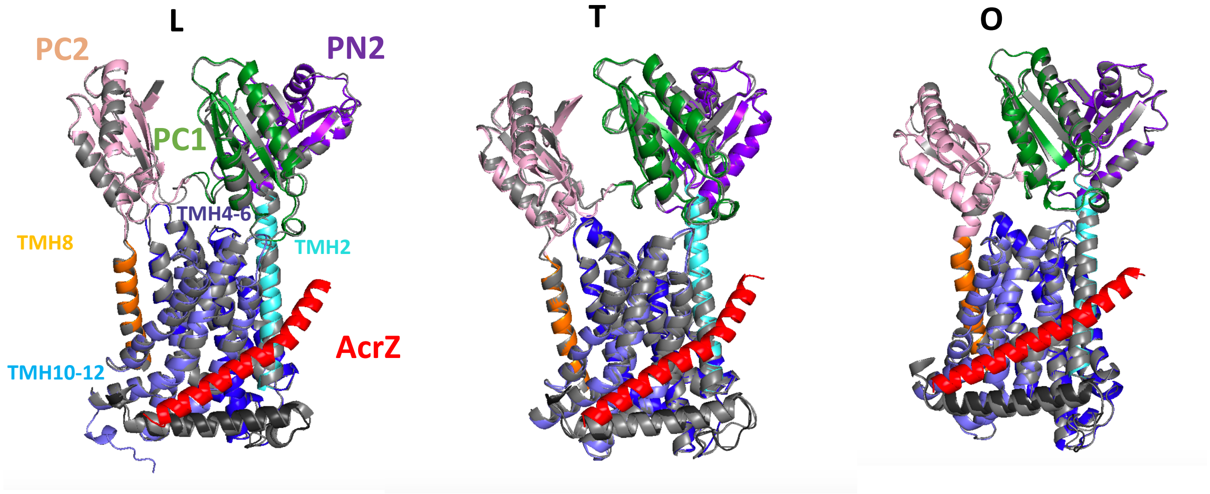

Figure S2. Related to Figure 3. Structural changes associated with AcrZ binding. Overlay of AcrB (grey; partially transparent) and AcrBZ (colored) protomers in L, T, O, conformation from cryo-EM derived structures of protein(s) reconstituted in *E. coli* lipids inside a saposin A-disc. To facilitate visualisation, only part of the protein is displayed for which changes were greatest: PC1/2, PN2, I2, TMH 2, TMH 8, TMH 10-12, and TMH 4-6 (i.e. reference frame). Color Code: PC2, pink; PN2, purple; PC1, dark green; TMH 8, orange; TMH 4-6, navy blue; TMH 2, cyan; TMH 10-12, blue; I2, grey.

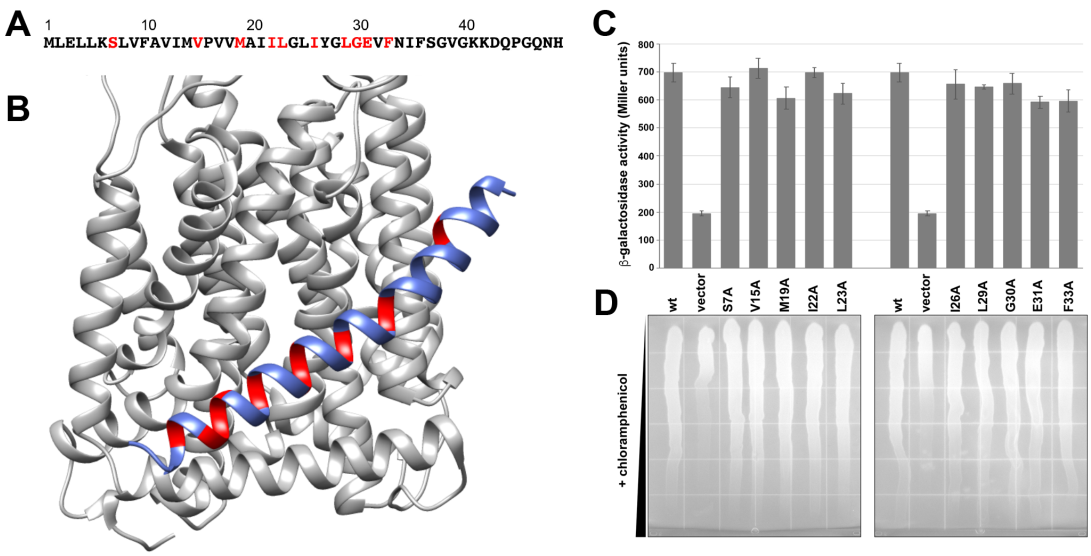

Figure S3. Related to Figure 4. Mutation of AcrZ interfacial residues. (**A**) The sequence of AcrZ. Interfacial residues predicted by previous structure and mutated are highlighted in red. (**B**) Structure of AcrB (grey) and AcrZ (blue) with interfacial residues indicated in red. (**C**) Split adenylate cyclase two-hybrid assays of the interaction between plasmid-encoded T25-AcrB and the empty vector, wild type AcrZ-T18 or the indicated mutant. T25-AcrB and the AcrZ-T18 indicated were co-expressed in an adenylate cyclase deficient strain and grown to OD_{600} ~ 1 when cells were harvested for β-galactosidase activity assay. Shown are the average and standard deviation of three experiments. The first and second wt and vector samples are the same. (**D**) Exponentially-growing cultures of the *E. coli* Δ*acrZ* strains carrying the pBAD24 empty vector, wild type AcrZ or the indicated AcrZ mutant were applied across chloramphenicol gradient plates to visualize differences in antibiotic sensitivity. The plates were incubated overnight at 37°C and photographed. Shown here is a representative image of an experiment carried out in triplicate.

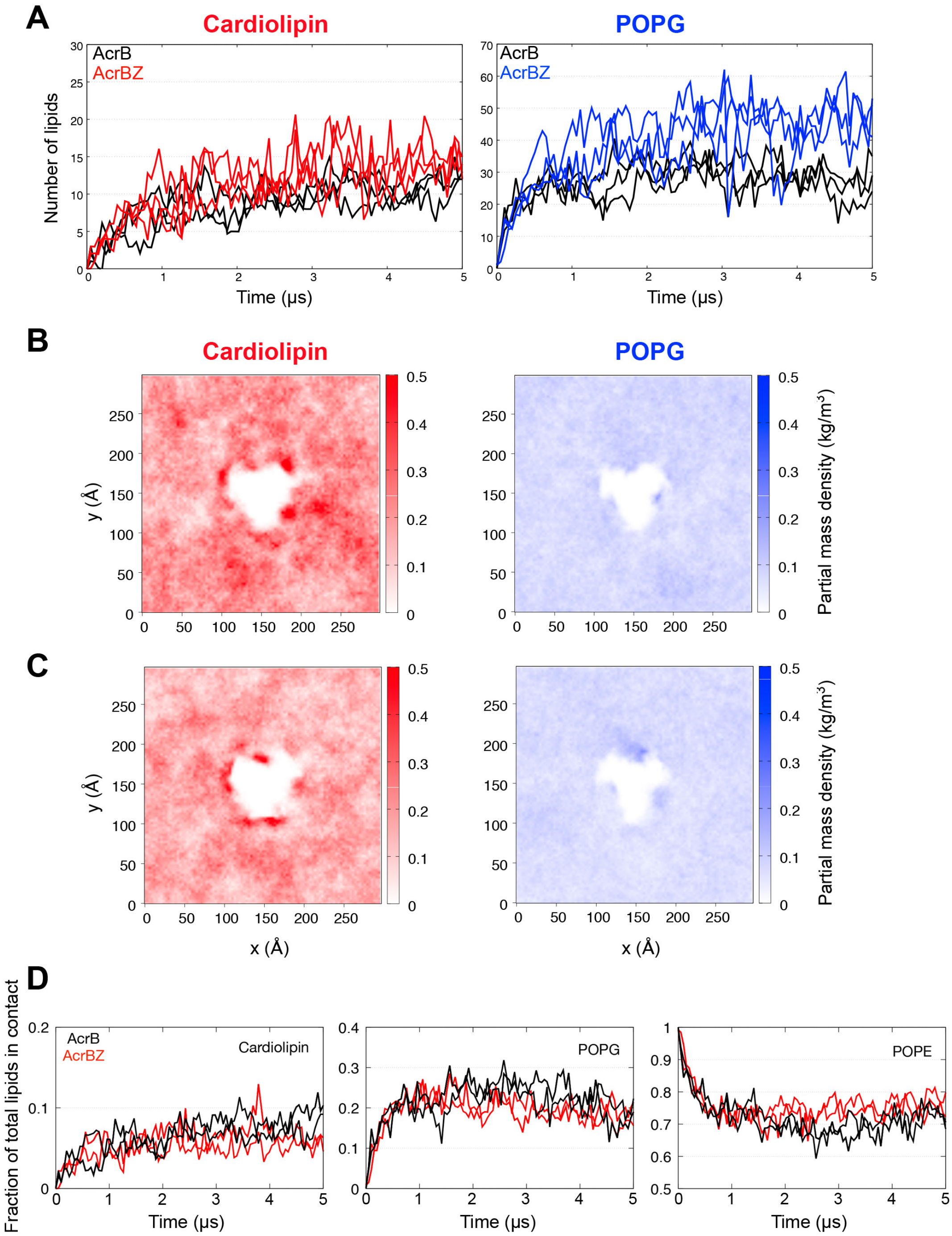

Figure S4. Related to Figure 5. Cardiolipin and POPG enrichment around AcrB and AcrBZ. (**A**) The number of cardiolipin (left) and POPG (right) found within 6 Å of AcrB (black) and AcrBZ (red or blue) throughout 5 μs of coarse-grained simulations. Data shown from three independent repeats. (**B**) Partial mass density of cardiolipin (red) and POPG (blue) and around AcrB (white space in the middle of the graph). This is normalized to the number of cardiolipin and POPG in the membrane. (**C**) Same

analysis performed on AcrBZ. (**D**) The number of each lipid species divided by the total number of lipids in contact with AcrB and AcrBZ during the simulation.

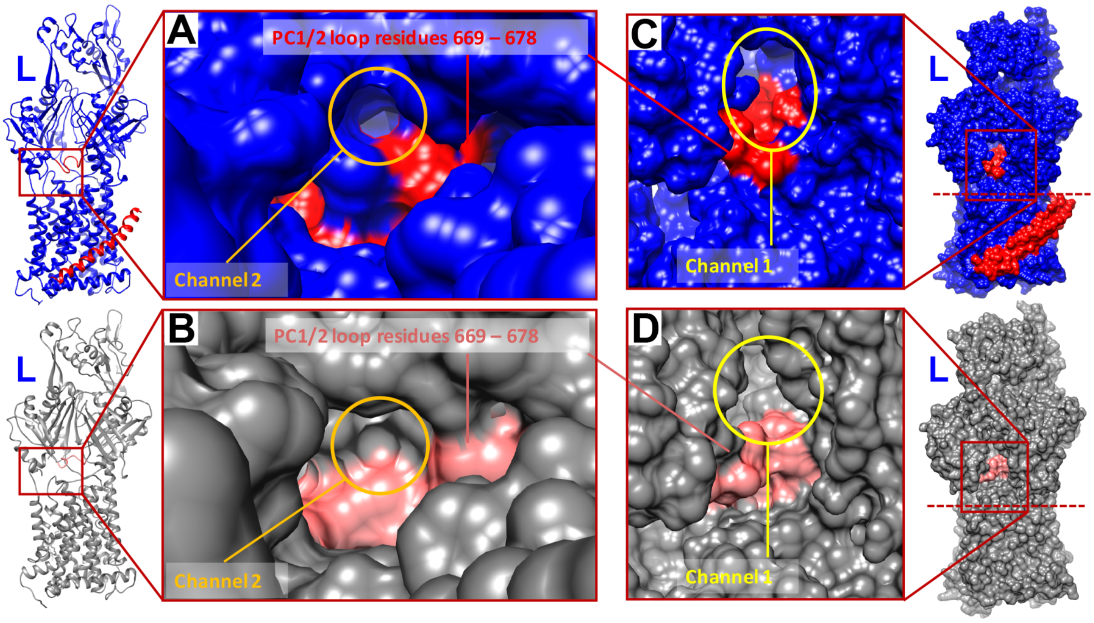

Figure S5. Related to Figure 6. Structural comparison of substrate entry site between saposin A disc reconstituted AcrB and AcrBZ with cardiolipin enrichment. L protomers of cryo-EM derived AcrB (in grey; partially transparent) reconstituted in *E. coli* lipids inside a saposin A disc and AcrBZ (blue) reconstituted in *E. coli* lipids enriched with cardiolipin inside a saposin A disc. Channel 2 entry is restricted by a loop region of PC1/2 (red) for substrate entry from the outer leaflet of the inner membrane. In the surface view, the entry site is open in case of the AcrBZ complex with 5% cardiolipin addition (**A**) and closed in AcrB without cardiolipin enrichment (**B**). The channel 1 entry site, protruding from the periplasm, is slightly lowered in case of AcrBZ (**C**), for easier access from above the membrane outer surface area, and elevated in case of AcrB (**D**). The dashed lines indicate the approximate level of the outer leaflet boundary with the periplasm (at which substrates presumably enter the complex).

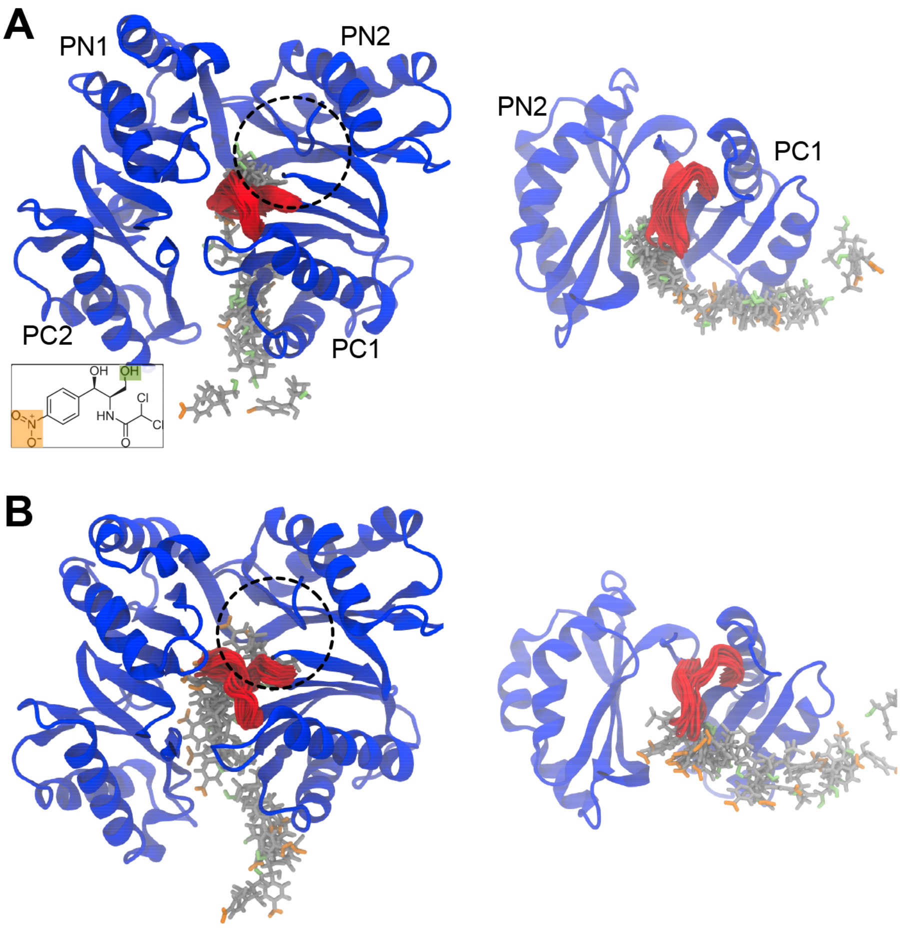

Figure S6. Related to Figure 6. Translocation of chloramphenicol into the binding site of AcrBZ and AcrB unaffected by the switch loop conformation. (**A**) A steered molecular dynamic simulation was performed whereby a chloramphenicol molecule was pulled from the periplasmic space into the deep binding pocket of the L protomer of AcrBZ. Views from the top (left) and side (right) of the protein are shown. Snapshots of chloramphenicol (nitrite group is orange, hydroxyl is green, and the rest is grey as shown in inset) and the switch loop (red) were taken every 1 ns of the simulation. The approximate position of the deep binding pocket is indicated by the black dotted circle. (**B**) A steered molecular dynamic simulation performed for AcrB showing a similar entry pathway of the drug into the protein. These simulations indicate that the switch loop is flexible and is able to allow the passage of chloramphenicol for both AcrBZ and AcrB.

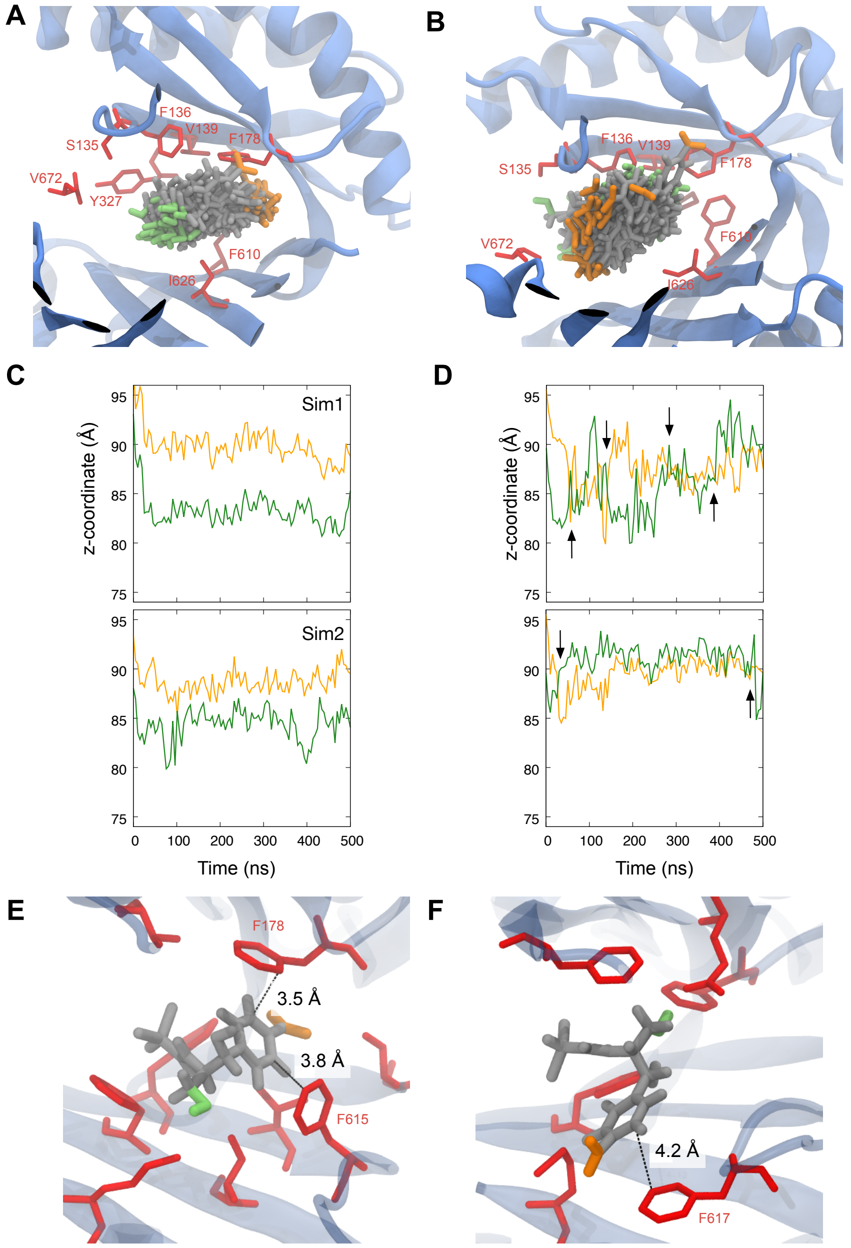

Figure S7. Related to Figure 6. Chloramphenicol binds more stably to AcrBZ with 5% cardiolipin. (**A**) and (**B**) Snapshots of chloramphenicol (same color scheme as Figure S6 for the antibiotic and Figure 6C,D for protein) bound to the binding site of the T protomer of AcrBZ and AcrB, respectively, taken every 10 ns from a 500 ns simulation. Residues highlighted in Figure 6C,D are shown in red. (**C**) and (**D**) The coordinate along the z-axis (perpendicular to the plane of the membrane) of the nitrite and hydroxyl moieties during two independent simulations (Sim1 and Sim2) with

AcrBZ and AcrB, respectively. Arrows in (**D**) indicate the time points at which the drug molecule flipped its orientation within the binding site. (**E**) and (**F**) The most frequently sampled conformation of the drug molecule from these simulations. The binding of chloramphenicol to AcrBZ was potentially stabilized by a pi-stacking interaction between its nitrobenzene ring and either F178 and F615. This interaction was absent in the AcrB simulations and the closest aromatic residue was F617, which was more than 4 Å away.

Table S1. Cryo-EM data collection and refinement statistics for AcrBZ and AcrB structures, Related to Figure 1.

**Structure**	AcrBZ saposin A disc Chloramphenicol	AcrB saposin A disc Minocycline	AcrBZ saposin A disc + 5% cardiolipin Chloramphenicol	AcrB saposin A disc + 5% cardiolipin Minocycline
PDB code	6SGS	6SGU	6SGR	6SGT
EMDB code	10183	10185	10182	10184
**Data collection**				
Microscope	FEI Titan Krios	FEI Titan Krios	FEI Titan Krios	FEI Titan Krios
Voltage (kV)	300	300	300	300
Detector	Falcon III	K2	Falcon III	Falcon III
Mode	Counting	Counting	Counting	Counting
Nominal magnification	75,000 ×	130,000 ×	75,000 ×	75,000 ×
Pixel size (Å)	1.09	1.06	1.09	1.06
Electron dose, per frame (e^{-}/Å^2)	0.350	1.335	0.586	0.919
Electron dose, total (e^{-}/Å^2)	26.25	53.4	46.9	44.1
Defocus range (µm), step (µm)	–1.3 to –3.4, 0.3	–1.3 to –3.4, 0.3	–1.3 to –3.4, 0.3	–1.3 to –3.4, 0.3
Exposure (s)	60	20	60	70
Frames	75	40	80	48
Number of micrographs	1,356	2,156	2,720	580
**Reconstruction**				
Software	RELION-3.0	RELION-3.0	RELION-3.0	RELION-3.0
Number of particles used	94,507	414,786	95,775	77,593
Final resolution, $FSC_{0.143}$ (Å)	3.20	3.27	3.17	3.46
Map-sharpening B factor (Å^2)	–107.0	–101.2	–99.6	–109.1
**Model composition**				
Non-hydrogen atoms	54,156	52,169	54,150	52,180
Protein residues	3,528	3,407	3,528	3,408
**Refinement**				
Software	Phenix-1.15.2	Phenix-1.15.2	Phenix-1.15.2	Phenix-1.15.2
Correlation coefficient, masked	0.82	0.82	0.83	0.79
Correlation coefficient, box	0.65	0.71	0.69	0.65
**Validation (proteins)**				
MolProbity score	1.12	1.39	1.19	1.49
Clash score, all atoms	2.03	3.26	2.14	3.28
EMRinger score	3.39	1.65	3.52	2.47
**Ramachandran plot statistics**				
Favoured, overall (%)	97.21	96.03	96.78	95.38
Allowed, overall (%)	2.79	3.94	3.22	4.53
Outlier, overall (%)	0.00	0.03	0.00	0.09
**R.m.s. deviations**				
Bond length (Å)	0.003	0.004	0.003	0.003
Bond angle (°)	0.581	0.634	0.533	0.633

Table S3. Oligonucleotides. Related to Figure 4.

JK1 (Fwd QuikChange Lightning Primer *acrZ* P16G)	CTGGTATTCGCCGTAATCATGGTAgggGTCGTGATGGCCA
JK2 (rev QuikChange Lightning Primer *acrZ* P16G)	TGGCCATCACGACcccTACCATGATTACGGCGAATACCAG
JK3 (Fwd QuikChange Lightning Primer *acrZ* V15G P16G)	GGTATTCGCCGTAATCATgggtggTGTCGTGATGGCCATCATCC
JK4 (rev QuikChange Lightning Primer *acrZ* V15G P16G)	GGATGATGGCCATCACGACAccacccATGATTACGGCGAATACC
JK5 (Fwd QuikChange Lightning Primer *acrZ* P16G V17G)	CTGGTATTCGCCGTAATCATGGTAggggggGTGATGGCCATCATCCTGG
JK6 (Rev QuikChange Lightning Primer *acrZ* P16G V17G)	CCAGGATGATGGCCATCACccccccTACCATGATTACGGCGAATACCAG
lr11 (fwd Q5 mutagenesis primer *acrZ* P16A A20P)	CGTCGTGATGcctATCATCCTGG
lr12 (rev Q5 mutagenesis primer *acrZ* P16A A20P)	GCTACCATGATTACGGCG
lr19 (fwd Q5 mutagenesis primer *acrZ* P16A V17P)	CATGGTAGCCcctGTGATGGCCATC
lr20 (rev Q5 mutagenesis primer *acrZ* P16A V17P)	ATTACGGCGAATACCAGAC
lr21 (fwd Q5 mutagenesis primer *acrZ* P16A V18P)	GGTAGCCGTCcctATGGCCATCATC
lr22 (rev Q5 mutagenesis primer *acrZ* P16A V18P)	ATGATTACGGCGAATACC
lr23 (fwd Q5 mutagenesis primer *acrZ* P16A M19P)	AGCCGTCGTGcctGCCATCATCC
lr24 (rev Q5 mutagenesis primer *acrZ* P16A M19P)	ACCATGATTACGGCGAATAC
lr40 (fwd QuikChange Lightning primer *acrZ* M19P)	CATGGTACCTGTCGTGccgGCCATCATCCTGGGTC
lr41 (rev QuikChange Lightning primer *acrZ* M19P)	GACCCAGGATGATGGCcggCACGACAGGTACCATG
lr46 (fwd QuikChange Lightning primer *acrZ* I26A)	ATGGCCATCATCCTGGGTCTGgcgTACGGTCTTGGTGAAGTATTC
lr47 (rev QuikChange Lightning primer *acrZ* I26A)	GAATACTTCACCAAGACCGTAcgcCAGACCCAGGATGATGGCCAT
lr50 (fwd Q5 mutagenesis primer *acrZ* A20P)	TGTCGTGATGcctATCATCCTGG
lr51 (rev Q5 mutagenesis primer *acrZ* A20P)	GGTACCATGATTACGGCG
mo20 (fwd Q5 mutagenesis primer *acrZ* S7A)	GTTATTAAAAgcactaGTATTCGCCGTAATCATG
mo21 (rev Q5 mutagenesis primer *acrZ* S7A)	TCTAACATCAAGCTTGGC
mo26 (fwd Q5 mutagenesis primer *acrZ* V15A)	CGTAATCATGgcaccgGTCGTGATGG
mo27 (rev Q5 mutagenesis primer *acrZ* V15A)	GCGAATACCAGACTTTTTAATAAC
mo30 (fwd Q5 mutagenesis primer *acrZ* M19A)	ACCTGTCGTGgccGCCATCATCC
mo31 (rev Q5 mutagenesis primer *acrZ* M19A)	ACCATGATTACGGCGAATAC
mo32 (fwd Q5 mutagenesis primer *acrZ* I22A)	TCGTGATGGCgatcgcCCTGGGTCTG
mo33 (rev Q5 mutagenesis primer *acrZ* I22A)	CAGGTACCATGATTACGG

mo34 (fwd Q5 mutagenesis primer *acrZ* L23A)	GGCCATCATCgcaGGTCTG ATTTACG
mo35 (rev Q5 mutagenesis primer *acrZ* L23A)	ATCACGACAGGTACCATG
mo45 (fwd Q5 mutagenesis primer *acrZ* L29A)	CGGTCTTGGTgccGTATTCA ACATC
mo46 (rev Q5 mutagenesis primer *acrZ* L29A)	TAAATCAGACCCAGGATG
mo47 (fwd Q5 mutagenesis primer *acrZ* G30A)	gccGAAGTATTCAACATCTT TTCTGG
mo48 (rev Q5 mutagenesis primer *acrZ* G30A)	TAGCCCGTAAATCAGACCC AGG
mo49 (fwd Q5 mutagenesis primer *acrZ* E31A)	CGGTCTTGGTgccGTATTCA ACATC
mo50 (rev Q5 mutagenesis primer *acrZ* E31A)	TAAATCAGACCCAGGATG
mo51 (fwd Q5 mutagenesis primer *acrZ* F33A)	TTGGTGAAGTggccAACATC TTTTCTGG
mo52 (rev Q5 mutagenesis primer *acrZ* F33A)	GACCGTAAATCAGACCCA G
mo67 (fwd Q5 mutagenesis primer *acrZ* P16A)	AATCATGGTAgccGTCGTGA TGG
mo68 (rev Q5 mutagenesis primer *acrZ* P16A)	ACGGCGAATACCAGACTT
