# Supplementary material for: Interactions of a Bacterial RND Transporter with a Transmembrane Small Protein in a Lipid Environment
Source: Structure. 2020 Jun 2;28(6):625–634.e6. doi: 10.1016/j.str.2020.03.013 (PMC7267776; doi:10.1016/j.str.2020.03.013)
Supplement: Document S2. Article plus Supplemental Information [file mmc3.pdf]

Structure

Article

Interactions of a Bacterial RND Transporter with a Transmembrane Small Protein in a Lipid Environment

Graphical Abstract

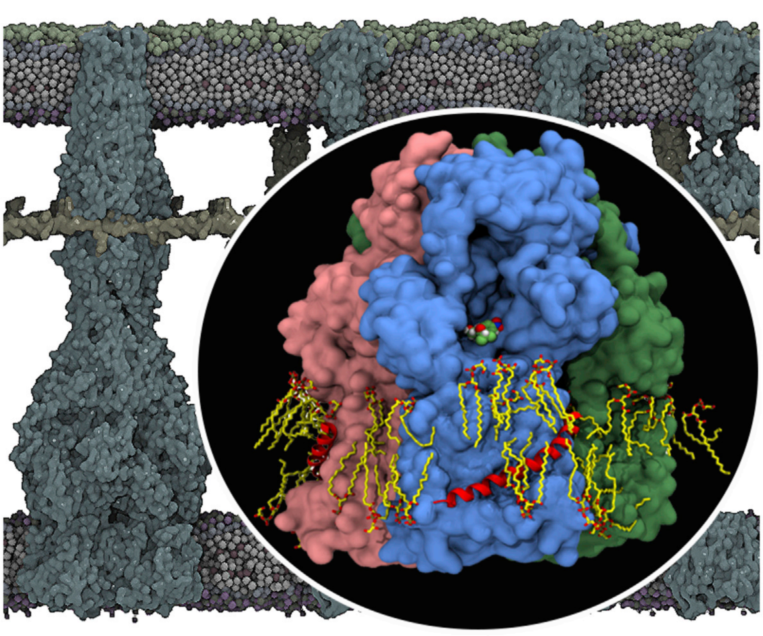

Lipids and small proteins modulate a tripartite efflux pump

Highlights

- Structure of an RND transporter with an allosteric modulator in a membrane environment
- Cooperation of lipid and small protein in allosterically modulating transport activity

Authors

Dijun Du, Arthur Neuberger, Mona Wu Orr, ..., Syma Khalid, Gisela Storz, Ben F. Luisi

Correspondence

dudj@shanghaitech.edu.cn (D.D.), s.khalid@soton.ac.uk (S.K.), storzg@mail.nih.gov (G.S.), bfl20@cam.ac.uk (B.F.L.)

In Brief

Multidrug efflux in bacteria contributes to their antibiotic resistance during host infection and is driven by transporters in the bacterial cell envelope. In this study, the structure of a multidrug transporter was determined in an environment mimicking the natural membrane, which includes a small protein that modulates efflux activity.

Du et al., 2020, Structure *28*, 625–634
June 2, 2020 © 2020 The Authors. Published by Elsevier Ltd.
https://doi.org/10.1016/j.str.2020.03.013

CellPress

Structure

CellPress
OPEN ACCESS

Article

Interactions of a Bacterial RND Transporter with a Transmembrane Small Protein in a Lipid Environment

Dijun Du,[1,4,7,*] Arthur Neuberger,[1,5,7] Mona Wu Orr,[2,7] Catherine E. Newman,[1,6,7] Pin-Chia Hsu,[3] Firdaus Samsudin,[3] Andrzej Szewczak-Harris,[1] Leana M. Ramos,[2] Mekdes Debela,[1] Syma Khalid,[3,*] Gisela Storz,[2,*] and Ben F. Luisi[1,8,*]

[1]Department of Biochemistry, University of Cambridge, Tennis Court Road, Cambridge CB2 1GA, UK

[2]Division of Molecular and Cellular Biology, Eunice Kennedy Shriver National Institute of Child Health and Human Development, Bethesda, MD 20892-4417, USA

[3]School of Chemistry, University of Southampton, Southampton SO17 1BJ, UK

[4]Present address: School of Life Science and Technology, ShanghaiTech University, Shanghai, China

[5]Present address: Columbia University Irving Medical Center, New York, NY, USA

[6]Present address: Harvard Medical School, Cambridge, MA, USA

[7]These authors contributed equally

[8]Lead Contact

*Correspondence: dudj@shanghaitech.edu.cn (D.D.), s.khalid@soton.ac.uk (S.K.), storzg@mail.nih.gov (G.S.), bfl20@cam.ac.uk (B.F.L.)

https://doi.org/10.1016/j.str.2020.03.013

SUMMARY

The small protein AcrZ in *Escherichia coli* interacts with the transmembrane portion of the multidrug efflux pump AcrB and increases resistance of the bacterium to a subset of the antibiotic substrates of that transporter. It is not clear how the physical association of the two proteins selectively changes activity of the pump for defined substrates. Here, we report cryo-EM structures of AcrB and the AcrBZ complex in lipid environments, and comparisons suggest that conformational changes occur in the drug-binding pocket as a result of AcrZ binding. Simulations indicate that cardiolipin preferentially interacts with the AcrBZ complex, due to increased contact surface, and we observe that chloramphenicol sensitivity of bacteria lacking AcrZ is exacerbated when combined with cardiolipin deficiency. Taken together, the data suggest that AcrZ and lipid cooperate to allosterically modulate AcrB activity. This mode of regulation by a small protein and lipid may occur for other membrane proteins.

INTRODUCTION

Numerous small proteins, corresponding to 100 or fewer codons, are encoded by phylogenetically diverse organisms and are likely to play key roles in many fundamental biological processes (Storz et al., 2014). Some small proteins have been discovered to regulate activity of large transport proteins, and in *Escherichia coli*, the 49-amino acid AcrZ modulates the action of AcrB, a homotrimeric secondary-active transporter that belongs to the resistance-nodulation-cell division (RND) superfamily (Hobbs et al., 2012). Deletion of the *acrZ* gene renders *E. coli* cells more sensitive to a subset of the antibiotics for which AcrB provides resistance (Hobbs et al., 2012). Transcription of *acrZ* is co-regulated with the *acrAB* operon, which also implicates the functional importance of the small protein for efflux activity (Hobbs et al., 2012).

AcrB is the energy-transducing component of a tripartite multidrug efflux machinery that includes the outer membrane protein TolC and the periplasmic bridging partner AcrA. Structures of the fully assembled tripartite complex together with AcrZ have been elucidated using cryoelectron microscopy (cryo-EM) (Du et al., 2014; Jeong et al., 2016; Wang et al., 2017b), revealing that the small protein forms a transmembrane helix which interacts extensively with the concave surface of AcrB in the transmembrane region. The interaction of AcrZ and AcrB *in situ* has been corroborated by mass spectrometry of the intact complex ejected directly from native membranes of *E. coli* cells (Chorev et al., 2018).

Although its influence over AcrB remains unclear from the available data, AcrZ was hypothesized to alter the conformation of the drug-binding pockets during the transport cycle and so change drug specificity. In this model, AcrZ could exert an influence on AcrB by changing the shape of the surface that is exposed to the lipid from a concave to a convex curvature, thus potentially affecting the interactions with lipids and distribution of lateral forces of the bilayer that can be communicated into the core of the transporter. In support for this proposal, experimental findings in other systems indicate that lipids and the membrane composition can have profound effects on structure, oligomerization, and activity of membrane proteins (Bechara et al., 2015; Gupta et al., 2017; Laganowsky et al., 2014). Moreover, a recent cryo-EM study of AcrB extracted directly from membranes reveals a semi-crystalline lipid organization within the central region of the transmembrane domains of the AcrB trimer that may support quaternary state transitions required for the transport mechanism (Qiu et al., 2018).

To investigate how AcrZ affects AcrB, we determined cryo-EM structures of AcrB and the AcrBZ complex reconstituted in a disc

This is an open access article under the CC BY license (http://creativecommons.org/licenses/by/4.0/).

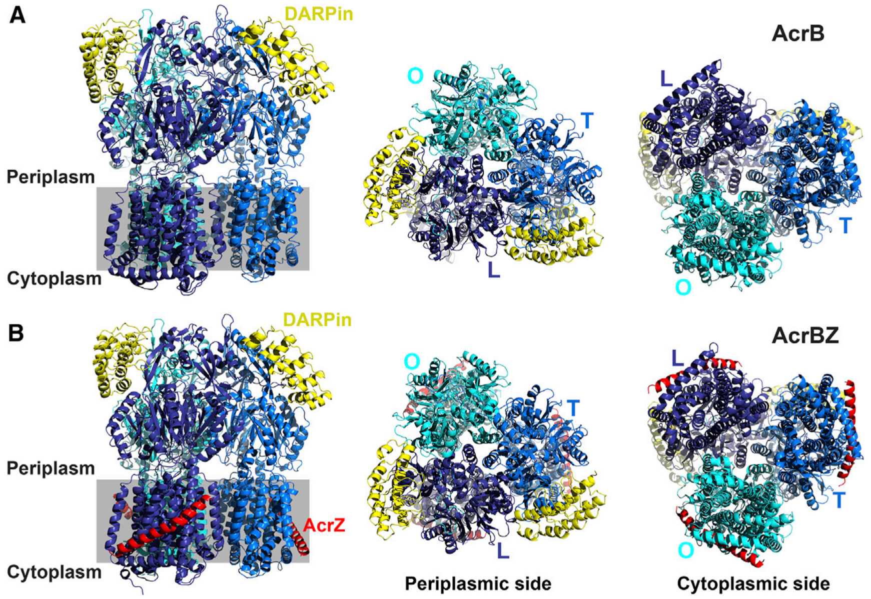

Figure 1. *E. coli* AcrB and AcrBZ in Saposin A Discs

(A) Structure of AcrB in saposin A discs prepared using *E. coli* lipids. Side (left; oriented so that the 3-fold axis is aligned vertically), periplasmic (center; along the 3-fold axis), and cytoplasmic (right; along the 3-fold axis) view. Within the trimer, the AcrB subunits are found in loose (L, navy blue), tight (T, blue), and open (O, cyan blue) conformations. Two DARPin domains (yellow) are positioned at the top of the periplasmic domain; attached to protomers in loose (L) and tight (T) conformations. The gray box indicates position of inner membrane.

(B) Structure of AcrBZ in saposin A discs in three views (see above). Two DARPin domains (yellow) are attached to protomers in L and T. AcrZ is displayed in red.

in which a bilayer of *E. coli* lipids is encircled by the membrane scaffold protein saposin A (Frauenfeld et al., 2016). To facilitate particle alignments for 3D reconstructions, the complexes included an engineered DARPin protein that binds the periplasmic domain of AcrB (Eicher et al., 2012; Sennhauser et al., 2006). Trimeric AcrB cycles through three states in the transport process, and DARPin associates with the subunits in the loose (L) and tight (T) states, but not with the periplasmic region of the open state (O). These three states can be observed in the cryo-EM reconstructions, enabling analysis of the AcrZ interactions with each state. We conclude that the combination of AcrZ and lipid environment work synergistically to provide an allosteric effect on the conformation of AcrB, with functional consequences for the dynamic substrate transport process.

RESULTS

Cryo-EM of DARPin-Bound AcrB and AcrBZ Reconstituted into Saposin A Discs

We developed a procedure to reconstitute purified *E. coli* AcrB and the AcrBZ complex into discs using *E. coli* lipids and saposin A as scaffolding protein (details in the STAR Methods). The reconstituted specimens behaved well on size-exclusion chromatography and eluted with a Gaussian-shaped profile in buffer without detergent, which is otherwise required to keep the membrane proteins soluble. To facilitate particle alignment from cryo-EM images of these specimens, we included an engineered DARPin that binds the periplasmic domain of AcrB (Eicher et al., 2012; Sennhauser et al., 2006). The disc-reconstituted, DARPin-bound AcrB and AcrBZ samples yielded excellent quality particles on cryo-EM grids (Figures S1A and S1B). Analysis of the particles provided interpretable maps with resolutions near 3.2 Å based on Fourier shell correlations. Models could be built into the density and refined with good stereochemistry (Table S1). Top, bottom, and side views of AcrB and AcrBZ are shown in Figure 1.

Saposin A monomers, which form a ring around the transmembrane domain of AcrB and the AcrBZ complex, were also resolved at a lower density, indicating that the ring is associated flexibly with the transporter. The features of the saposin A become more diffuse as refinement progressed, while the features of the AcrB and AcrBZ become sharper. A lipid layer could be visualized in the maps, and acyl chains or lipid head groups were included in the refined models.

A well-organized lipid bilayer within the central region of the transmembrane domains of the AcrB trimer was noted in earlier studies of specimens solubilized with styrene-maleic acid copolymers (Qiu et al., 2018). We also observe a lipid layer in our maps in the corresponding region (Figure 2A). Our best model for the AcrBZ complex in saposin A discs is consistent with acyl chain packing observed in the structure of AcrB in co-polymers (Figure 2A). Simulations show that the acyl chains on the inner leaflet pack in a stable lattice with approximate hexagonal geometry (Figure 2B), visible also in our cryo-EM maps, while they form a less regular pattern in the outer leaflet. The calculations indicate that acyl chains are more mobile in the outer layer compared with the inner layer (Figure 2C). Hydrophobic side chains of AcrB are predicted to interact with the acyl groups of these lipids in different ways for the three protomers (Figure 2D); such differences could help to communicate conformational signals between the subunits associated with transitions between the L, T, and O states, as proposed earlier (Qiu et al., 2018). Density is also present for lipids on the outer surface where AcrZ interacts, but the lipids here are not extensively ordered.

The Lipid Environment Affects the Orientation of AcrZ on AcrB

The models of AcrB and AcrBZ in the saposin discs were compared against previously described crystal structures of detergent-solubilized AcrB and AcrBZ, respectively (Figure 3). As a reference frame for the comparison, we used transmembrane helices (TMH) 4-6, which had previously been demonstrated to be a suitable group for overlays due to its conformationally invariance in the L, T, and O states (Murakami et al., 2006; Seeger et al., 2006). Small conformational shifts were detected between the detergent-based crystal structure and the saposin disc-reconstituted cryo-EM structure for both AcrB and AcrBZ, including a rotation of the periplasmic domain

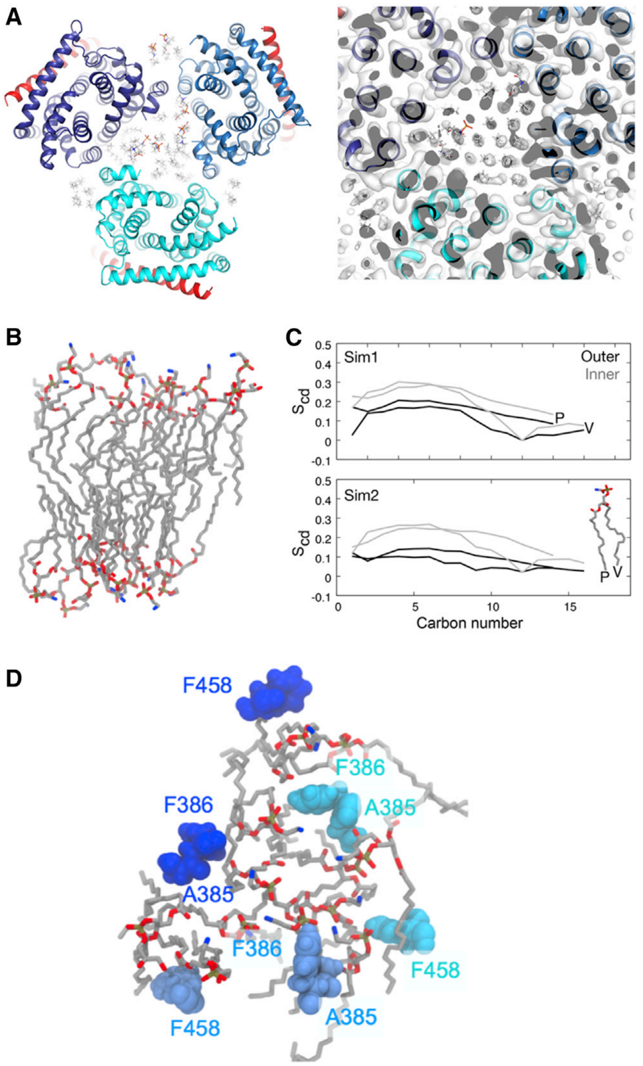

Figure 2. Organization and Interactions of Lipids in the Central Cavity of AcrB

(A) Left panel shows a view along the central axis of the AcrBZ complex from the cytoplasmic side, with *E. coli* lipids at natural abundance. Right panel shows the cryo-EM density in the central region, which reveals the hexagonal pattern for the lipids. Colors of AcrB subunits and AcrZ are the same as in Figure 1.

(B) Snapshots of the lipid bilayer in the central cavity of AcrB at the end of a 500-ns simulation.

(C) Average order parameters (S_{cd}) of the palmitic (P) and vaccenic (V) acid lipid tails of PVPE (1-palmitoyl 2-cis-vaccenic phosphatidylethanolamine) in the outer and inner leaflets of the central cavity in two simulations (Sim1 and Sim2).

(D) Hydrophobic residues that protrude into the outer leaflet of the lipid bilayer during the simulations in the different AcrB protomers, viewed perpendicular to the plane of the membrane (L, navy blue; T, blue; and O, cyan blue).

(foremost in PC2, PN2, and PC1 sub-domains) in all three states (L, T, and O). These differences in the periplasmic domain could reflect the response of the protein to constraints imposed or enforced by the crystal lattice. A structural comparison of AcrB and AcrBZ when both are in saposin A discs indicates small changes in all three AcrB protomers in PC1/2 and PN2 and TMH 2 and 8 (Figure S2). These changes may be a consequence of the presence of AcrZ.

In the AcrBZ structure in saposin A discs, we noted a previously unseen, significant bending of AcrZ toward the binding groove in AcrB, which was most pronounced in L and O states. Each of the three AcrZs in the saposin disc bends toward AcrB between residues 10 and 15 (F10, A11, V12, I13, M14, and V15) (Figures 4A and 4B). The bending mode is also predicted by molecular dynamics simulations of the AcrBZ complex in a lipid bilayer (Figure 4C). The bend could be caused by the presence of lipids in the saposin disc mediating new contacts with AcrB or otherwise exerting a force on AcrZ.

Defined Bend in AcrZ Important for Interaction with AcrB and Effect on Efflux

A proline at position 16 is expected to be a helix-breaker (Figure 4D), conferring a kink to the AcrZ protein due to the R-group ring. Given the significant bending seen in this region in the structural analysis, we asked whether the proline residue is important for the AcrZ interaction with AcrB. Using a two-hybrid approach with AcrB and AcrZ fused to the T25 and T18 fragments of *Bordetella pertussis* adenylate cyclase, respectively, we assayed for interactions that restore adenylate cyclase function, and consequently induce β-galactosidase activity (Hobbs et al., 2012). We first substituted an alanine for the proline residue (P16A) and observed that for this mutant the β-galactosidase activity was equivalent to empty vector levels, indicating that the P16A variant could not interact with AcrB (Figure 4E). We then shifted the location of the proline from position 16 to positions 17, 18, 19, and 20 (P16A V17P, P16A V18P, P16A M19P, and P16A A20P). With the exception of P16A V18P, all mutations restored β-galactosidase activity to wild-type levels (Figure 4E). We next examined the consequences of introducing a second proline at positions 19 and 20 (M19P, A20P). The M19P and A20P variants had reduced β-galactosidase compared with wild-type. Finally, we investigated the effect of changing flexibility in this region by introducing single and double glycine substitutions (P16G, V15G P16G, and P16G V17G). In principle, glycine could permit the AcrZ to bend in the same conformation favored by proline, and indeed all glycine substitution AcrZ variants were able to interact with AcrB at wild-type levels (Figure 4E). Together, these indicate that a bend in AcrZ around position 16 is important for the AcrZ-AcrB interaction.

To investigate the effects of AcrZ mutations on drug efflux, untagged derivatives were expressed from a plasmid in a Δ*acrZ* strain background and assayed for resistance to chloramphenicol. Compared with the vector control, wild-type *acrZ* provided increased resistance to chloramphenicol on gradient plates (Figure 4F). As expected, mutant AcrZ variants unable to interact with AcrB were unable to rescue chloramphenicol resistance. However, several AcrZ mutants capable of interacting with AcrB were nonetheless unable to restore growth to wild-type levels (V15G P16G; P16G V17G). Interestingly, two mutants that had a wild-type or intermediate phenotype for AcrB interaction (P16A M19P; M19P) showed reduced chloramphenicol resistance to below that of the empty vector, while two other mutants with a wild-type AcrB interaction (P16A A20P; A20P) were more resistant than the wild-type AcrZ strain.

Mutations of individual interfacial residues to alanine did not have a strong effect on either the AcrZ interaction with AcrB or chloramphenicol resistance (Figure S3). Together, these indicate

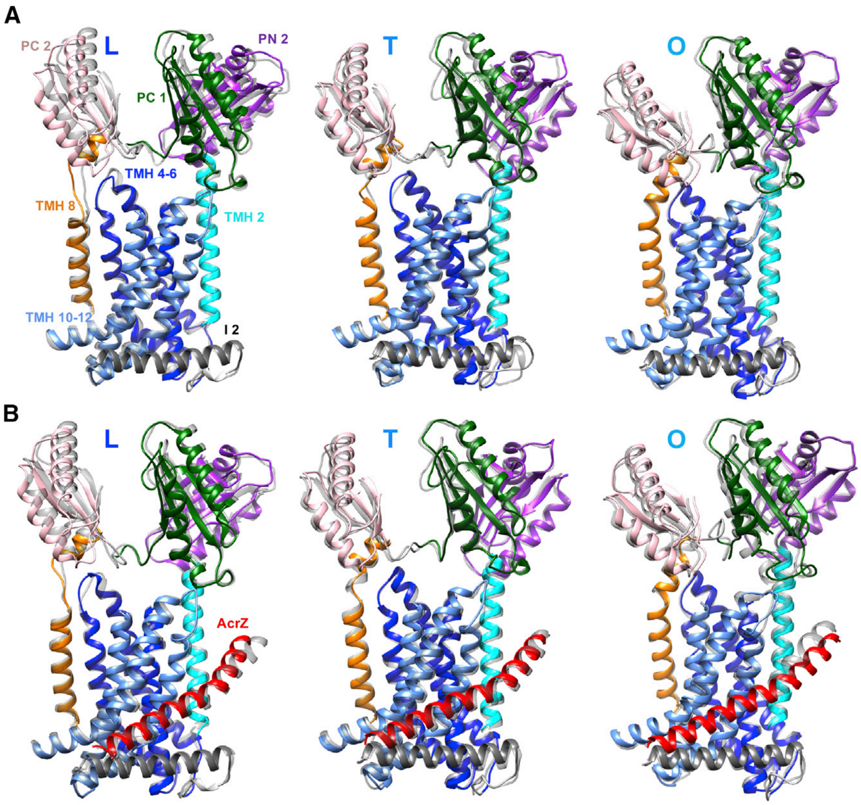

Figure 3. Gallery of Structural Comparisons of AcrB and AcrBZ Crystal Structures (Gray) Overlaid by Cryo-EM Saposin A Nanodisc Structures (Colored)

(A) Overlay of protomers in L, T, and O of AcrB crystal structure (PDB: 4DX5) (light gray; partially transparent) and cryo-EM-derived AcrB structure reconstituted in *E. coli* lipids inside a saposin A nanodisc. Orientations of protomer overlays L, T, and O in space were adjusted in depiction for a better display of observed changes. Color code: PC2, pink; PN2, purple; PC1, dark green; TMH 8, orange; TMH 4-6, navy blue; TMH 2, cyan blue; TMH 10-12, blue; I2, gray. Displayed are PC1/2, PN2, I2, TMH 2, TMH 8, TMH 10-12, and TMH 4-6 (i.e., reference frame).

(B) Overlay of protomers in L, T, and O of (1) AcrBZ crystal structure (PDB: 5NC5) (in gray; partially transparent) and (2) cryo-EM-derived AcrBZ structure reconstituted in *E. coli* lipids inside a saposin A nanodisc. The overlays in both (A) and (B) were produced using MatchMaker command in Chimera using the rigid TMH 4-6 (blue) of AcrB crystal structure as reference frame.

that the overall hydrophobic character and bent shape of AcrZ, rather than specific AcrB-AcrZ contacts, are important for physical interactions. Moreover, the mutations also indicate that direct interactions of AcrB-AcrZ are necessary but not sufficient for efflux function, and that the interactions can either suppress or support pump activity depending on context. These features are a hallmark of an allosteric system.

Lipid Interactions with AcrB and AcrBZ

It had previously been suggested that lipids can modulate the structure and function of specific membrane proteins, and cardiolipin has been identified as such a modulator (Bechara et al., 2015; Gupta et al., 2017; Laganowsky et al., 2014). We carried out coarse-grained molecular dynamics simulations of AcrB and AcrBZ with an *E. coli* cytoplasmic membrane model composed of mostly palmityloleoyl phosphatidylethanolamine (POPE), but also including cardiolipin and palmityloleoyl phosphatidylglycerol (POPG), which are the least abundant lipids in the natural cytoplasmic membrane. Our simulations started with no cardiolipin and POPG molecules within 30 Å of the protein, but after 5 μs as many as 15 cardiolipin and 50 POPG molecules were found within 6 Å of the protein (Figure S4). The cardiolipin and POPG enriched around both proteins despite the membrane containing a smaller number of these lipid types compared with POPE. A slightly higher degree of enrichment was observed around AcrBZ compared with AcrB, especially in the case of POPG. Analysis of density plots suggest that the interaction between these lipids with AcrB or AcrBZ may be non-specific, as high-density regions were found around all parts of the transmembrane portion of the proteins (Figure S4). The higher number of cardiolipin and POPG for AcrBZ is likely to result from the increased protein surface area (Figure S4D). Because it is easier to genetically manipulate the cardiolipin composition of the *E. coli* inner membrane, we focused our functional and structural studies on this special lipid.

The *E. coli* inner membrane has been reported to contain around 5% cardiolipin (Dowhan, 1997), with some localized enrichment in negatively curved regions of the membrane, such as the cell poles (Renner and Weibel, 2011). To test the effects of cardiolipin on the conformation of AcrB and AcrBZ, we reconstituted the proteins into saposin discs in which the *E. coli* lipids were supplemented with an additional 5% cardiolipin (i.e., to ~10% total abundance). The increase in the cardiolipin content is associated with small changes in PC1/2 and PN2 for AcrB (Figure S2). Minor changes were seen when comparing the AcrBZ complexes in the presence and absence of excess cardiolipin. The greatest changes were seen in comparing AcrB with natural lipids with AcrBZ in the lipid environment with added cardiolipin (Figure S2).

Additive Effects of AcrZ and Cardiolipin for Growth Viability and Drug Binding

E. coli lacking AcrZ was previously shown to be less resistant to chloramphenicol compared with the wild-type parental strain (Hobbs et al., 2012). Because the conformational changes in AcrB observed in the presence of AcrZ and additional cardiolipin, we asked whether cardiolipin could affect chloramphenicol resistance. Thus, the wild-type strain (*E. coli* MG1655) and strains lacking AcrZ (MG1655 Δ*acrZ*), cardiolipin (MG1655 Δ*clsABC*:FRT-*kan*-FRT), or both (MG1655 Δ*clsABC*::FRT-*kan*-FRT Δ*acrZ*) were assayed for chloramphenicol resistance. As reported previously,

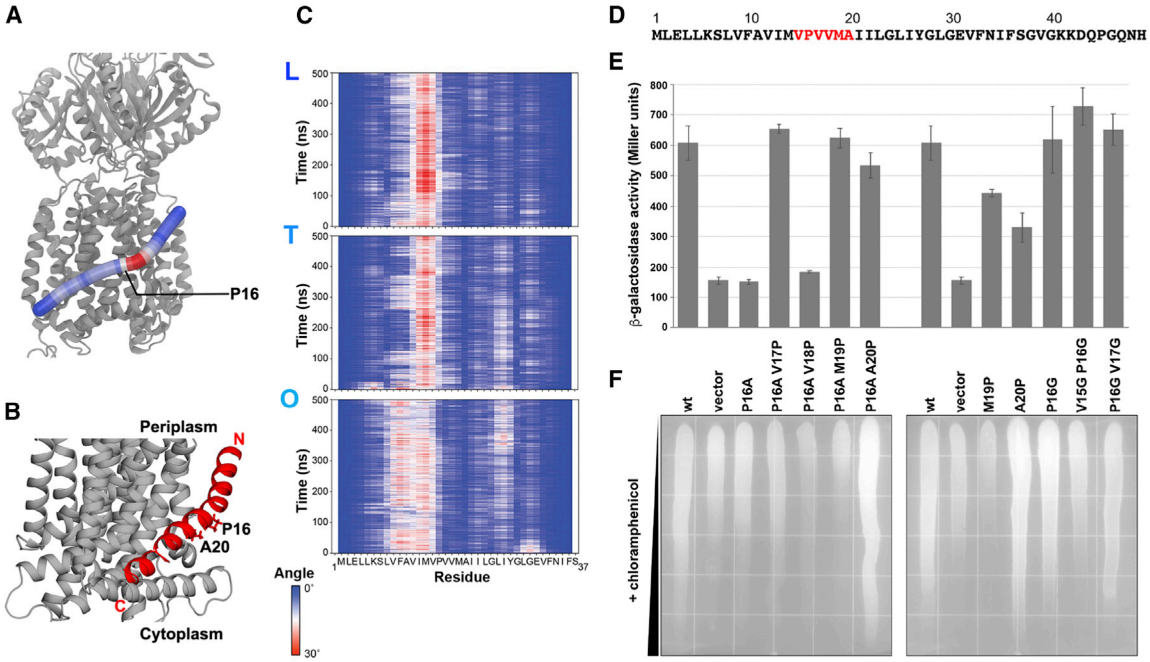

Figure 4. Impact of AcrZ Curvature on Binding to AcrB

(A) A snapshot of AcrZ bound to the AcrB in the loose conformation. The AcrZ is shown in tube representation and colored based on the bending angle between its residues, from blue (0°) to red (30°), while the AcrB is shown in ribbon representation and colored gray.

(B) A zoom in of AcrZ bound to AcrB. AcrZ is oriented with the N terminus in the periplasm and the C terminus in the cytoplasm. The locations of P16 and A20 are indicated.

(C) The evolution of the bending angle over the course of one of the simulations for all three AcrZ subunits. The conformation of AcrB (L, T, O) to which these AcrZ subunits are bound is shown on top left of each graph.

(D) The sequence of AcrZ with residues mutated indicated in red.

(E) Split adenylate cyclase two-hybrid assays of the interaction between plasmid-encoded T25-AcrB and the empty vector, wild-type AcrZ-T18 or the indicated mutant. T25-AcrB and the AcrZ-T18 indicated were co-expressed in an adenylate cyclase-deficient strain and grown to optical density at 600 nm (OD_{600}) ~ 1 when cells were harvested for β-galactosidase activity assay. Shown are the average and standard deviation of three experiments. The first and second wild-type (wt) and vector samples are the same.

(F) Exponentially growing cultures of the *E. coli* Δ*acrZ* strains carrying the pBAD24 empty vector, wild-type AcrZ, or the indicated AcrZ mutant were applied across chloramphenicol gradient plates to visualize differences in antibiotic sensitivity. The plates were incubated overnight at 37°C and photographed. Shown here is a representative image of an experiment carried out in triplicate.

the *acrZ* deletion strain showed increased sensitivity to chloramphenicol but not erythromycin (Hobbs et al., 2012) (Figure 5). Strains deficient for cardiolipin were also more sensitive to chloramphenicol. Strikingly, the double-mutant strain was most sensitive to chloramphenicol, consistent with the largest structural changes being seen in the presence of both AcrZ and cardiolipin. These indicate that AcrZ and cardiolipin have additive effects on AcrB ability to export chloramphenicol (Figure 5).

Combination of AcrZ and Cardiolipin Is Associated with Changes in the AcrB Entry Channels, Gating Loop, and Binding Pocket

Efflux of substrates through AcrB is a complex process given that AcrB has multiple entry channels and multiple binding sites that are used by different types of drugs (reviewed in Zwama and Yamaguchi, 2018). Deletion of *acrZ* affects only a subset of the antibiotics effluxed by AcrB. The mechanism behind this specificity is unknown, but it is possible that AcrZ selectively affects certain entry channels or binding locations. Thus, we examined structural differences in AcrB regions involved in drug transport in the presence and absence of AcrZ and cardiolipin. For the AcrB set we used minocycline as ligand, and for AcrBZ we used chloramphenicol. We found that AcrZ and cardiolipin affect both of the substrate entry channels (Figures 6A and S5). Channel 1, for entry from the periplasm, has an altered entry shape with closer access from above the membrane surface in AcrBZ compared with AcrB. Channel 2, which protrudes sideward from above the outer leaflet of the membrane into the protomer in the L state, is more restricted by a loop region part of PC1/2 of the AcrB structure in a saposin disc without cardiolipin enrichment when compared with AcrBZ structure in the saposin disc with ~10% cardiolipin.

There are also changes in the switch loop that could influence the passage of the drug from the L to the T states (Figure 6B). To explore the process of substrate movement through the pocket, molecular dynamics simulations were conducted in which a chloramphenicol molecule was pulled from the periplasmic space into the deep-binding pocket of the L protomer of AcrB and AcrBZ with 10% cardiolipin. These simulations suggest that the movement of the antibiotic is unaffected by the switch loop at the entry gate of the ligand from the L to the T state due to its inherent flexibility (Figure S6).

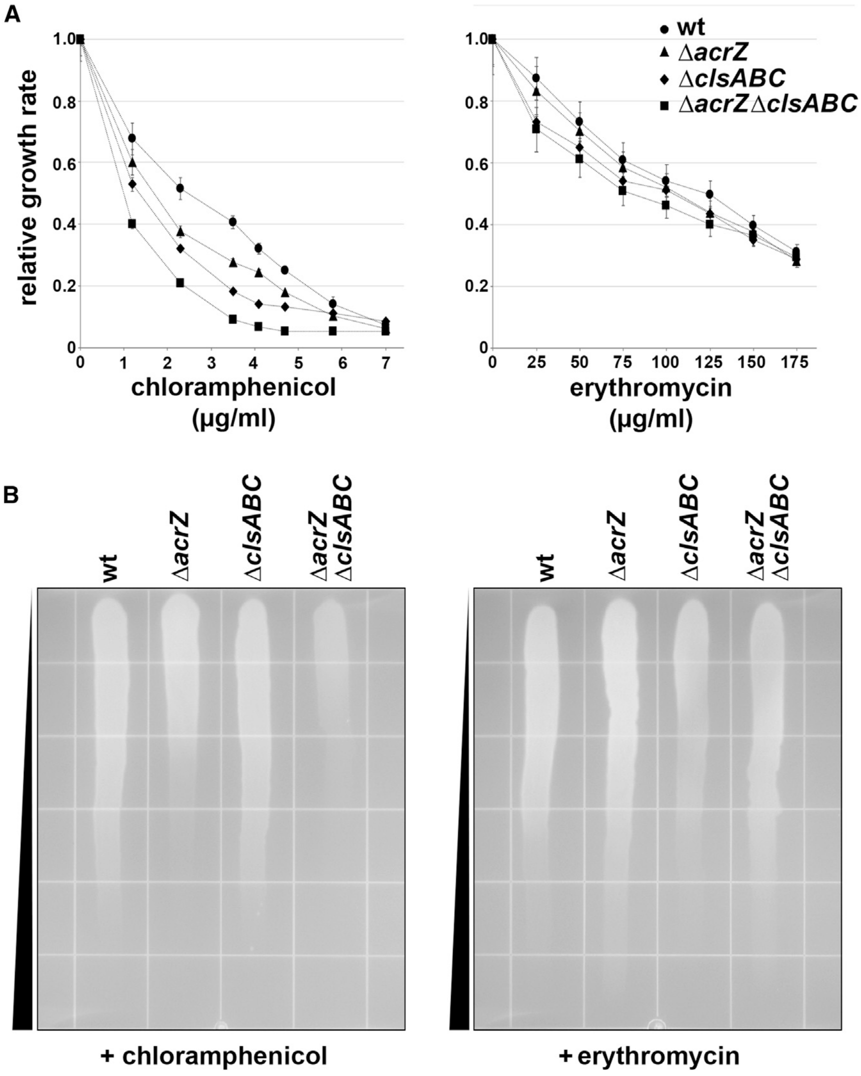

Figure 5. Cardiolipin Biogenesis and AcrZ Impact on Chloramphenicol Sensitivity

(A) Growth rates of *E. coli* MG1655 (wild-type parent strain), MG1655 Δ*acrZ*, MG1655 Δ*clsABC*::FRT-*kan*-FRT (cardiolipin-deficient), and MG1655 Δ*clsABC*::FRT-*kan*-FRT Δ*acrZ* (cardiolipin-deficient and Δ*acrZ*) in the presence of a range of chloramphenicol (0–7 µg mL^{-1}) and erythromycin (0–175 µg mL^{-1}) concentrations were determined relative to the maximum growth rate in the absence of drug. OD_{660} measurements are presented as mean of triplicate measurements ± standard error of the mean. For the determination of the relative growth rates of the cultures in each of the wells, the exponential phase of the growth curve (as a mean of n = 3 for each culture type) was determined from the linear increase in a $log_{10}(OD_{660})$ versus time plot. The slope of this section was determined by simple linear regression. Heteroscedasticity-consistent standard errors of the corresponding slope coefficient were calculated. The quality of the fit was significant in all cases ($p < 0.05$). Next, the relative growth rate was determined as the ratio of the growth rate in the presence of drug over the maximum growth rate in the absence of drug.

(B) Exponentially growing cultures of the above strains were applied across chloramphenicol or erythromycin gradient plates to visualize differences in antibiotic sensitivity. The plates were incubated overnight at 37°C and photographed. Shown is a representative gradient plate image of a biological replicate from experiments carried out for ten different single colonies. The deletion of chromosomal *acrZ* gave a reproducible but slight increase in resistance to erythromycin in this *E. coli* strain background, but this mild effect is not observed in a different *E. coli* strain background (Hobbs et al., 2012).

The observed changes in PC1/2 and PN2 of AcrB are likely to affect drug-binding pocket properties in the T protomer given the orientation of key residues for substrate binding (Figure 6C). When AcrBZ saposin discs with supplementary cardiolipin were prepared with the addition of chloramphenicol, additional density was found in the cryo-EM map in the distal pocket of the AcrB protomer in the T state, which could be easily fitted with a molecule of the antibiotic (Figure 6D). The structural changes we observe are unlikely due to incubation with different substrates (minocycline versus chloramphenicol), because we do not observe density in the T or L pockets for either minocycline (in AcrB ± cardiolipin with saposin A) or chloramphenicol (in the AcrBZ complex without additional cardiolipin with saposin A). We also tested chloramphenicol with one of the preparations of AcrB in saposin A discs but did not see the ligand (not shown). The chloramphenicol only becomes visible in the T state pocket in the case of AcrBZ with additional cardiolipin. These data suggest that the presence of AcrZ and cardiolipin can influence the occupancy of the substrate in the binding pocket.

Molecular dynamics simulations also indicate that chloramphenicol is bound more stably to the binding site of the T protomer of the AcrBZ +10% cardiolipin structure compared with the AcrB structure (Figure S7). Chloramphenicol significantly and frequently changed its orientation and failed to reach a stable conformation in the simulations of AcrB. Together, the structures and simulations show that, although the effects of AcrZ and cardiolipin on the transport activity of AcrB might not occur at the substrate encounter with the switch loop, multiple other steps are affected.

DISCUSSION

The development of a procedure for efficient reconstitution of AcrBZ into saposin A scaffold-based discs with a native lipid environment, suitable for cryo-EM analysis and with an option to alter the lipid content, revealed previously undescribed structural differences in the conformation of AcrZ. The structure, together with mutagenesis data, revealed that the overall bent

CellPress
OPEN ACCESS

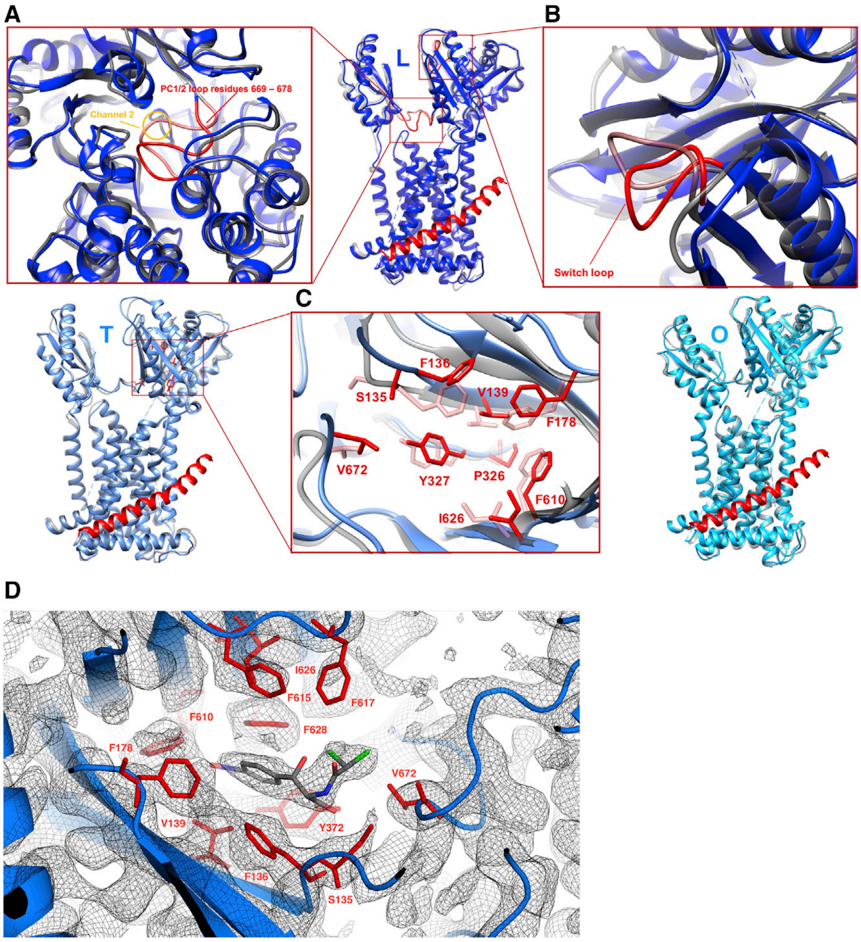

Figure 6. Structural Comparison Between Saposin A Disc-Reconstituted AcrB and AcrBZ with Cardiolipin Enrichment

Overlay of protomers in L, T, O of cryo-EM-derived AcrB (in gray; partially transparent) reconstituted in *E. coli* lipids inside a saposin A disc and AcrBZ (blue variants) reconstituted in *E. coli* lipids enriched with cardiolipin inside a saposin A disc.

(A) Channel 2 entry is restricted by a loop region of PC1/2 (light red) for substrate entry from the outer leaflet of the inner membrane in case of AcrB in L state but open in the case of the AcrBZ complex in L (dark red).

(B) Once substrate enters the protomer in L, a switch loop (red) allows passage into the deep-binding pocket in AcrB in complex with AcrZ. This loop appears to restrict access in case of the AcrB L protomer in the absence of AcrZ.

(C) Impact on the drug-binding pocket at the site of chloramphenicol binding. Chloramphenicol should be located inside the distal pocket of the AcrB in "tight" conformation. Upon inspection of this area, no discernible chloramphenicol density could be identified, but the antibiotic is predicted to pack against residues P326, Y327, V139, F136, F610, F178, S135, I626, and V672 (red side chains). The residues are mostly on two sets of beta sheets in the porter domain of AcrB, as well as on nearby loops.

(D) Cryo-EM density in the T state pocket for the AcrBZ complex with additional cardiolipin, showing the binding of chloramphenicol (gray) in a discrete conformation, with surrounding residues highlighted. Even though chloramphenicol or minocycline was added to other samples as well, density was not observed in this position for the maps for the AcrBZ and AcrB structures in the natural lipid composition or AcrB with cardiolipin supplement.

shape of AcrZ is being recognized rather than specific interacting residues, which also may be the case for the transmembrane interactions of other small proteins with their cognate partners (Hobson et al., 2018). The elucidation of a more native conformation of both AcrB and the AcrBZ complex helps to explain the mechanism behind the resistance-modulating effect of AcrZ binding to AcrB for a subset of its substrates.

In light of the discovery of AcrZ, there appears to be a crucial yet unexplored general role of small proteins for modulating activity of efflux pumps. For example, in muscle cells, a group of small proteins regulate calcium uptake by the SERCA pump (Anderson et al., 2015). In *E. coli*, KdpF is another small protein that increases the activity of a potassium transport protein (KdpFABC), while SgrT is a small protein that inhibits the glucose transport activity of EIICB (Storz et al., 2014). Structural studies of the roles of these small proteins can further elucidate the different mechanisms of possible allosteric modulation. A number of other small proteins detected in *E. coli* are also found at the membrane (Hemm et al., 2008), and we speculate that many of these proteins can act as allosteric modulators of target membrane protein partners.

Accumulating evidence indicates that lipids can affect the localization, structure, stability, and function of certain membrane proteins (Bechara et al., 2015; Gupta et al., 2017; Laganowsky et al., 2014; Renner and Weibel, 2011). Lipids have, for example, been shown to directly regulate opening of the mechanosensitive channel MscS (Pliotas et al., 2015). *E. coli* aquaporin Z was found to be stabilized and functionally modulated by cardiolipin, and the *E. coli* ammonia channel AmtB was shown to selectivity bind phosphatidylglycerol (Laganowsky et al., 2014). Another study with co-polymer-solubilized AcrB identified a pattern of lipid organization around the transmembrane portion (Qiu et al., 2018). We observe lipids in our maps as well, and our best model for the AcrBZ complex with lipids is consistent with acyl chain packing and lipid head group interactions with AcrB observed in the other study (Figure 2).

We structurally, computationally, and functionally explored the impact of cardiolipin on AcrBZ. Taken together, our data suggest that the interaction of AcrZ and AcrB increases affinity of the complex for cardiolipin- and POPG-enriched environments inside the inner membrane. This effect does not appear to be due to the formation of specific interactions, but instead may

originate from the greater surface for lipid contact in the presence of the AcrZ subunit (Figure S4). Cardiolipin and other lipids, together with AcrZ, modulate the activity of AcrB through allosteric changes—putatively by inducing structural alterations in the drug entry and binding sites. Our cryo-EM maps suggest that cardiolipin and AcrZ together also seem to encourage a more discrete binding mode for chloramphenicol in the AcrB distal pocket. This could be a reflection of, for example, an increased specificity for the drug when both regulators are present. The activities of other bacterial and mitochondrial membrane proteins have also been reported to be affected by interactions with cardiolipin (Dudek, 2017). Both a small protein and cardiolipin have been separately shown to promote the activity of MgtA, a magnesium importer in *E. coli* (Subramani et al., 2016; Wang et al., 2017a). Cardiolipin thus influences processes ranging from electron transport to antimicrobial resistance by affecting protein localization, enhancing protein stability, mediating interactions between monomer units, and transmitting conformation changes between subunits (Dudek, 2017). In addition, recent work has found that cardiolipin plays a role in proton motif force stimulation and modulation of ATPase activity in SecYEG (Corey et al., 2018). Cardiolipin also promotes the distribution of the osmosensory transporter ProP to the cellular poles in *E. coli* (Romantsov et al., 2007). Interestingly, in older bacterial cells, the AcrAB/TolC complex tends to cluster at the pole (Bergmiller et al., 2017), where it potentially could encounter a distinct lipid environment with impact on its function and activity. The recent studies of protein-lipid interactions, and cardiolipin in particular, show the importance of continued study of membrane proteins in their native lipid environment. Knowledge of these interactions may impact on our understanding the mechanism of drug resistance in clinical treatment.

STAR★METHODS

Detailed methods are provided in the online version of this paper and include the following:

- KEY RESOURCES TABLE
- LEAD CONTACT AND MATERIALS AVAILABILITY
- EXPERIMENTAL MODEL AND SUBJECT DETAILS
- METHOD DETAILS
  - Preparation of Saposin A
  - Protein Expression, Purification and Nanodisc Reconstitution
  - Electron Cryo-Microscopy
  - Image Processing and 3D Reconstruction
  - Model Docking and Refinement
  - Molecular Dynamics Simulations
  - Bacterial Two-Hybrid Assays
  - Gradient Plate Assay
  - Cell Growth for Drug Sensitivity Assay
- QUANTIFICATION AND STATISTICAL ANALYSIS
- DATA AND CODE AVAILABILITY

SUPPLEMENTAL INFORMATION

Supplemental Information can be found online at https://doi.org/10.1016/j.str.2020.03.013.

ACKNOWLEDGMENTS

The work was supported by an ERC advanced award (742210) and by a Wellcome Trust Investigator award to B.F.L. C.E.N. was supported by a Gates-Cambridge Scholarship and A.N. by a Herchel Smith Research Studentship. Work by L.M.R., M.W.O., and G.S. was supported by the Intramural Program of the Eunice Kennedy Shriver National Institute of Child Health and Human Development. We acknowledge Diamond for access and support of the cryo-EM facilities at the UK National Electron Bio-Imaging Centre (eBIC), funded by the Wellcome Trust, MRC, and BBSRC. We thank colleagues at eBIC, especially Dan Clare and Alistair Siebert, for help with cryo-EM data collection. We thank Dima Chirgadze for help with data collection using the University of Cambridge cryo-EM facility. We also thank Douglas B. Weibel for kindly providing strains deficient in cardiolipin biogenesis and Jacob Olondo Kuba for help generating some AcrZ mutants. We are extremely grateful to Tristan Croll for advice and help with ISOLDE for the refinement of the models, and to Anirban Banerjee, Alex Sodt, Yvette Nstogo and Malitha Ratnaweera for helpful and insightful discussions.

AUTHOR CONTRIBUTIONS

D.D., A.N., M.W.O., C.E.N., P.-C.H., F.S., A.S.-H., L.M.R., M.D., S.K., G.S., and B.F.L. planned the studies. D.D., A.N., M.W.O., C.E.N., P.-C.H., F.S., A.S.-H., L.M.R., M.D., and S.K. carried out the studies. D.D., A.N., M.W.O., C.E.N., P.-C.H., F.S., A.S.-H., L.M.R., M.D., S.K., G.S., and B.F.L. carried out the data analysis and prepared the manuscript.

DECLARATION OF INTERESTS

The authors declare that they have no conflict of interest.

Received: December 8, 2019
Revised: February 14, 2020
Accepted: March 27, 2020
Published: April 28, 2020

REFERENCES

Abraham, M.J., Murtola, T., Schulz, R., Páll, S., Smith, J.C., Hess, B., and Lindahl, E. (2015). GROMACS: high performance molecular simulations through multi-level parallelism from laptops to supercomputers. SoftwareX *1*, 19–25.

Adams, P.D., Afonine, P.V., Bunkóczi, G., Chen, V.B., Davis, I.W., Echols, N., Headd, J.J., Hung, L.-W., Kapral, G.J., and Grosse-Kunstleve, R.W. (2010). PHENIX: a comprehensive Python-based system for macromolecular structure solution. Acta Crystallogr. D Biol. Crystallogr. *66*, 213–221.

Aibara, S., Kato, M., Ishinaga, M., and Kito, M. (1972). Changes in positional distribution of fatty acids in the phospholipids of *Escherichia coli* after shift-down in temperature. Biochim. Biophys. Acta *270*, 301–306.

Anderson, D.M., Anderson, K.M., Chang, C.-L., Makarewich, C.A., Nelson, B.R., McAnally, J.R., Shelton, J.M., Liou, J., Bassel-Duby, R., and Olson, E.N. (2015). Regulation of muscle contractility by a family of SERCA-inhibitory micropeptides. Cell *160*, 595–606.

Barad, B.A., Echols, N., Wang, R.Y.-R., Cheng, Y., DiMaio, F., Adams, P.D., and Fraser, J.S. (2015). EMRinger: side chain-directed model and map validation for 3D cryo-electron microscopy. Nat. Methods *12*, 943.

Bechara, C., Nöll, A., Morgner, N., Degiacomi, M.T., Tampé, R., and Robinson, C.V. (2015). A subset of annular lipids is linked to the flippase activity of an ABC transporter. Nat. Chem. *7*, 255–262.

Berendsen, H.J., van Postma, J., van Gunsteren, W.F., DiNola, A., and Haak, J. (1984). Molecular dynamics with coupling to an external bath. J. Chem. Phys. *81*, 3684–3690.

Bergmiller, T., Andersson, A.M., Tomasek, K., Balleza, E., Kiviet, D.J., Hauschild, R., Tkačik, G., and Guet, C.C. (2017). Biased partitioning of the multidrug efflux pump AcrAB-TolC underlies long-lived phenotypic heterogeneity. Science *356*, 311–315.

Bryson, V., and Szybalski, W. (1952). Microbial selection. Science *116*, 45–51.

CellPress
OPEN ACCESS

Bussi, G., Donadio, D., and Parrinello, M. (2007). Canonical sampling through velocity rescaling. J. Chem. Phys. *126*, 014101.

Castillo, N., Monticelli, L., Barnoud, J., and Tieleman, D.P. (2013). Free energy of WALP23 dimer association in DMPC, DPPC, and DOPC bilayers. Chem. Phys. Lipids *169*, 95–105.

Chen, V.B., Arendall, W.B., Headd, J.J., Keedy, D.A., Immormino, R.M., Kapral, G.J., Murray, L.W., Richardson, J.S., and Richardson, D.C. (2010). MolProbity: all-atom structure validation for macromolecular crystallography. Acta Crystallogr. D Biol. Crystallogr. *66*, 12–21.

Chorev, D.S., Baker, L.A., Wu, D., Beilsten-Edmands, V., Rouse, S.L., Zeev-Ben-Mordehai, T., Jiko, C., Samsudin, F., Gerle, C., and Khalid, S. (2018). Protein assemblies ejected directly from native membranes yield complexes for mass spectrometry. Science *362*, 829–834.

Corey, R.A., Pyle, E., Allen, W.J., Watkins, D.W., Casiraghi, M., Miroux, B., Arechaga, I., Politis, A., and Collinson, I. (2018). Specific cardiolipin–SecY interactions are required for proton-motive force stimulation of protein secretion. Proc. Natl. Acad. Sci. U S A *115*, 7967–7972.

Croll, T.I. (2018). ISOLDE: a physically realistic environment for model building into low-resolution electron-density maps. Acta Crystallogr. D Struct. Biol. *74*, 519–530.

Dahl, A.C.E., Chavent, M., and Sansom, M.S. (2012). Bendix: intuitive helix geometry analysis and abstraction. Bioinformatics *28*, 2193–2194.

DeLano, W.L. (2002). The PyMOL Molecular Graphics System. http://www.pymol.org.

Dowhan, W. (1997). Molecular basis for membrane phospholipid diversity: why are there so many lipids? Annu. Rev. Biochem. *66*, 199–232.

Du, D., Wang, Z., James, N.R., Voss, J.E., Klimont, E., Ohene-Agyei, T., Venter, H., Chiu, W., and Luisi, B.F. (2014). Structure of the AcrAB-TolC multidrug efflux pump. Nature *509*, 512–515.

Dudek, J. (2017). Role of cardiolipin in mitochondrial signaling pathways. Front. Cell Dev. Biol. *5*, 90.

Eicher, T., Cha, H.-j., Seeger, M.A., Brandstätter, L., El-Delik, J., Bohnert, J.A., Kern, W.V., Verrey, F., Grütter, M.G., and Diederichs, K. (2012). Transport of drugs by the multidrug transporter AcrB involves an access and a deep binding pocket that are separated by a switch-loop. Proc. Natl. Acad. Sci. U S A *109*, 5687–5692.

Emsley, P., Lohkamp, B., Scott, W.G., and Cowtan, K. (2010). Features and development of Coot. Acta Crystallogr. D Biol. Crystallogr. *66*, 486–501.

Essmann, U., Perera, L., Berkowitz, M.L., Darden, T., Lee, H., and Pedersen, L.G. (1995). A smooth particle mesh Ewald method. J. Chem. Phys. *103*, 8577–8593.

Frauenfeld, J., Löving, R., Armache, J.P., Sonnen, A.F., Guettou, F., Moberg, P., Zhu, L., Jegerschöld, C., Flayhan, A., Briggs, J.A., et al. (2016). A saposin-lipoprotein nanoparticle system for membrane proteins. Nat. Methods *13*, 345–351.

Gupta, K., Donlan, J.A., Hopper, J.T., Uzdavinys, P., Landreh, M., Struwe, W.B., Drew, D., Baldwin, A.J., Stansfeld, P.J., and Robinson, C.V. (2017). The role of interfacial lipids in stabilizing membrane protein oligomers. Nature *541*, 421–424.

Guzman, L.M., Belin, D., Carson, M.J., and Beckwith, J. (1995). Tight regulation, modulation, and high-level expression by vectors containing the arabinose PBAD promoter. J. Bacteriol. *177*, 4121–4130.

Hemm, M.R., Paul, B.J., Schneider, T.D., Storz, G., and Rudd, K.E. (2008). Small membrane proteins found by comparative genomics and ribosome binding site models. Mol. Microbiol. *70*, 1487–1501.

Hess, B., Bekker, H., Berendsen, H.J., and Fraaije, J.G. (1997). LINCS: a linear constraint solver for molecular simulations. J. Comput. Chem. *18*, 1463–1472.

Hobbs, E.C., Astarita, J.L., and Storz, G. (2010). Small RNAs and small proteins involved in resistance to cell envelope stress and acid shock in *Escherichia coli*: analysis of a bar-coded mutant collection. J. Bacteriol. *192*, 59–67.

Hobbs, E.C., Yin, X., Paul, B.J., Astarita, J.L., and Storz, G. (2012). Conserved small protein associates with the multidrug efflux pump AcrB and differentially affects antibiotic resistance. Proc. Natl. Acad. Sci. U S A *109*, 16696–16701.

Hobson, J.J., Gallegos, A.S., Atha, B.W., III, Kelly, J.P., Lein, C.D., VanOrsdel, C.E., Weldon, J.E., and Hemm, M.R. (2018). Investigation of amino acid specificity in the CydX small protein shows sequence plasticity at the functional level. PLoS One *13*, e0198699.

Hoover, W.G. (1985). Canonical dynamices: equilibrium phase-space distributions. Phys. Rev. A Gen. Phys. *31*, 1695–1697.

Huang, J., and MacKerell, A.D., Jr. (2013). CHARMM36 all-atom additive protein force field: validation based on comparison to NMR data. J. Comput. Chem. *34*, 2135–2145.

Humphrey, W., Dalke, A., and Schulten, K. (1996). VMD: visual molecular dynamics. J. Mol. Graph. *14*, 33–38.

Jeong, H., Kim, J.-S., Song, S., Shigematsu, H., Yokoyama, T., Hyun, J., and Ha, N.-C. (2016). Pseudoatomic structure of the tripartite multidrug efflux pump AcrAB-TolC reveals the intermeshing cogwheel-like interaction between AcrA and TolC. Structure *24*, 272–276.

Jo, S., Lim, J.B., Klauda, J.B., and Im, W. (2009). CHARMM-GUI membrane builder for mixed bilayers and its application to yeast membranes. Biophys. J. *97*, 50–58.

Kim, S., Lee, J., Jo, S., Brooks, C.L., III, Lee, H.S., and Im, W. (2017). CHARMM-GUI ligand reader and modeler for CHARMM force field generation of small molecules. J. Comput. Chem. *38*, 1879–1886.

Kucukelbir, A., Sigworth, F.J., and Tagare, H.D. (2014). Quantifying the local resolution of cryo-EM density maps. Nat. Methods *11*, 63–65.

Laganowsky, A., Reading, E., Allison, T.M., Ulmschneider, M.B., Degiacomi, M.T., Baldwin, A.J., and Robinson, C.V. (2014). Membrane proteins bind lipids selectively to modulate their structure and function. Nature *510*, 172–175.

Liebschner, D., Afonine, P.V., Baker, M.L., Bunkóczi, G., Chen, V.B., Croll, T.I., Hintze, B., Hung, L.-W., Jain, S., McCoy, A.J., et al. (2019). Macromolecular structure determination using X-rays, neutrons and electrons: recent developments in Phenix. Acta Crystallogr. D Struct. Biol. *D75*, 861–877.

Lins, R.D., and Hünenberger, P.H. (2005). A new GROMOS force field for hexopyranose-based carbohydrates. J. Comput. Chem. *26*, 1400–1412.

Ludtke, S.J., Baldwin, P.R., and Chiu, W. (1999). EMAN: semiautomated software for high-resolution single-particle reconstructions. J. Struct. Biol. *128*, 82–97.

Lugtenberg, E., and Peters, R. (1976). Distribution of lipids in cytoplasmic and outer membranes of *Escherichia coli* K12. Biochim. Biophys. Acta *441*, 38–47.

Miller, J.H. (1992). A Short Course in Bacterial Genetics: A Laboratory Manual and Handbook for *Escherichia coli* and Related Bacteria (Cold Spring Harbor Laboratory Press).

Monticelli, L., Kandasamy, S.K., Periole, X., Larson, R.G., Tieleman, D.P., and Marrink, S.-J. (2008). The MARTINI coarse-grained force field: extension to proteins. J. Chem. Theor. Comput. *4*, 819–834.

Murakami, S., Nakashima, R., Yamashita, E., Matsumoto, T., and Yamaguchi, A. (2006). Crystal structures of a multidrug transporter reveal a functionally rotating mechanism. Nature *443*, 173–179.

Nosé, S. (1984). A unified formulation of the constant temperature molecular dynamics methods. J. Chem. Phys. *81*, 511–519.

Oliver, P.M., Crooks, J.A., Leidl, M., Yoon, E.J., Saghatelian, A., and Weibel, D.B. (2014). Localization of anionic phospholipids in *Escherichia coli* cells. J. Bacteriol. *196*, 3386–3398.

Parrinello, M., and Rahman, A. (1981). Polymorphic transitions in single crystals: a new molecular dynamics method. J. Appl. Phys. *52*, 7182–7190.

Periole, X., Cavalli, M., Marrink, S.-J., and Ceruso, M.A. (2009). Combining an elastic network with a coarse-grained molecular force field: structure, dynamics, and intermolecular recognition. J. Chem. Theor. Comput. *5*, 2531–2543.

Pettersen, E.F., Goddard, T.D., Huang, C.C., Couch, G.S., Greenblatt, D.M., Meng, E.C., and Ferrin, T.E. (2004). UCSF Chimera—a visualization system for exploratory research and analysis. J. Comput. Chem. *25*, 1605–1612.

Pliotas, C., Dahl, A.C.E., Rasmussen, T., Mahendran, K.R., Smith, T.K., Marius, P., Gault, J., Banda, T., Rasmussen, A., and Miller, S. (2015). The role of lipids in mechanosensation. Nat. Struct. Mol. Biol. *22*, 991.

Qi, Y., Ingolfsson, H.I., Cheng, X., Lee, J., Marrink, S.J., and Im, W. (2015). CHARMM-GUI martini maker for coarse-grained simulations with the martini force field. J. Chem. Theor. Comput. *11*, 4486–4494.

Qiu, W., Fu, Z., Xu, G.G., Grassucci, R.A., Zhang, Y., Frank, J., Hendrickson, W.A., and Guo, Y. (2018). Structure and activity of lipid bilayer within a membrane-protein transporter. Proc. Natl. Acad. Sci. U S A *115*, 12985–12990.

Renner, L.D., and Weibel, D.B. (2011). Cardiolipin microdomains localize to negatively curved regions of *Escherichia coli* membranes. Proc. Natl. Acad. Sci. U S A *108*, 6264–6269.

Romantsov, T., Helbig, S., Culham, D.E., Gill, C., Stalker, L., and Wood, J.M. (2007). Cardiolipin promotes polar localization of osmosensory transporter ProP in *Escherichia coli*. Mol. Microbiol. *64*, 1455–1465.

Rosenthal, P.B., and Henderson, R. (2003). Optimal determination of particle orientation, absolute hand, and contrast loss in single-particle electron cryomicroscopy. J. Mol. Biol. *333*, 721–745.

Scheres, S.H. (2012). RELION: implementation of a Bayesian approach to cryo-EM structure determination. J. Struct. Biol. *180*, 519–530.

Seeger, M.A., Schiefner, A., Eicher, T., Verrey, F., Diederichs, K., and Pos, K.M. (2006). Structural asymmetry of AcrB trimer suggests a peristaltic pump mechanism. Science *313*, 1295–1298.

Sennhauser, G., Amstutz, P., Briand, C., Storchenegger, O., and Grütter, M.G. (2006). Drug export pathway of multidrug exporter AcrB revealed by DARPin inhibitors. PLoS Biol. *5*, e7.

Storz, G., Wolf, Y.I., and Ramamurthi, K.S. (2014). Small proteins can no longer be ignored. Annu. Rev. Biochem. *83*, 753–777.

Subramani, S., Perdreau-Dahl, H., and Morth, J.P. (2016). The magnesium transporter A is activated by cardiolipin and is highly sensitive to free magnesium in vitro. Elife *5*, https://doi.org/10.7554/eLife.11407.

Thomason, L.C., Costantino, N., and Court, D.L. (2007). *E. coli* genome manipulation by P1 transduction. Curr. Protoc. Mol. Biol. *79*, 1.17.11–11.17.18.

Wang, H., Yin, X., Wu Orr, M., Dambach, M., Curtis, R., and Storz, G. (2017a). Increasing intracellular magnesium levels with the 31-amino acid MgtS protein. Proc. Natl. Acad. Sci. U S A *114*, 5689–5694.

Wang, Z., Fan, G., Hryc, C.F., Blaza, J.N., Serysheva, I.I., Schmid, M.F., Chiu, W., Luisi, B.F., and Du, D. (2017b). An allosteric transport mechanism for the AcrAB-TolC multidrug efflux pump. Elife *6*, e24905.

Wolf, M.G., Hoefling, M., Aponte-Santamaría, C., Grubmüller, H., and Groenhof, G. (2010). g_membed: efficient insertion of a membrane protein into an equilibrated lipid bilayer with minimal perturbation. J. Comput. Chem. *31*, 2169–2174.

Yokota, K., Kanamoto, R., and Kito, M. (1980). Composition of cardiolipin molecular species in *Escherichia coli*. J. Bacteriol. *141*, 1047–1051.

Zhang, K. (2016). Gctf: Real-time CTF determination and correction. J. Struct. Biol. *193*, 1–12.

Zheng, S.Q., Palovcak, E., Armache, J.-P., Verba, K.A., Cheng, Y., and Agard, D.A. (2017). MotionCor2: anisotropic correction of beam-induced motion for improved cryo-electron microscopy. Nat. Methods *14*, 331.

Zivanov, J., Nakane, T., Forsberg, B.O., Kimanius, D., Hagen, W.J., Lindahl, E., and Scheres, S.H. (2018). New Tools for automated high-resolution cryo-EM structure determination in RELION-3. eLife *e42166*.

Zwama, M., and Yamaguchi, A. (2018). Molecular mechanisms of AcrB-mediated multidrug export. Res. Microbiol. *169*, 372–383.

STAR★METHODS

KEY RESOURCES TABLE

REAGENT or RESOURCE	SOURCE	IDENTIFIER
Bacterial and Virus Strains		
Escherichia coli adenylate cyclase deletion strain BTH101	Euromedex	N/A
E. coli MG1655 (wild type parent strain)	Storz laboratory	N/A
E. coli MG1655 Δ*acrZ*	Hobbs et al., 2012	GS0284
E. coli MG1655 Δ*clsABC*::FRT-*kan*-FRT (cardiolipin-deficient)	Douglas B. Weibel	N/A
E. coli MG1655 Δ*acrZ::kan*	Hobbs et al. 2012	GSO598
E. coli MG1655 Δ*acrZ*	This study	N/A
E. coli MG1655 Δ*clsABC*::FRT-*kan*-FRT (cardiolipin-deficient)	This study	N/A
E. coli MG1655 Δ*clsABC*::FRT-*kan*-FRT Δ*acrZ* (cardiolipin-deficient and Δ*acrZ*)	This study	N/A
E. coli shuffle-T7	New England Biolabs	C3029J
Chemicals, Peptides, and Recombinant Proteins		
E.coli lipid extract	Avanti	100500
cardiolipin	Avanti	841199
Ortho-nitrophenyl-beta-galactoside (ONPG)	Sigma	N1127-5G
Deposited Data		
AcrB in complex with AcrZ in saposin A disc and chloramphenicol	This study	PDB 6SGS<br>EMDB 10183
AcrB in complex with AcrZ in saposin A disc + supplementary cardiolipin and chloramphenicol	This study	PDB 6SGR<br>EMDB 10182
AcrB saposin A disc and minocycline	This study	PDB 6SGU<br>EMDB 10185
AcrB saposin A disc + supplementary cardiolipin and minocycline	This study	PDB 6SGT<br>EMDB 10184
AcrB with DARPin in detergent	Eicher et al., 2012	PDB 4DX5
AcrB in complex with AcrZ and puromycin in detergent	Wang et al., 2017b	PDB 5NC5
AcrB in complex with AcrZ in detergent	Du et al., 2014	PDB 4CDI
Oligonucleotides		
see Table S3		
Recombinant DNA		
pBAD24, arabinose-inducible expression vector	Guzman et al., 1995	N/A
pBAD24-*acrZ* from *E. coli* MG1655 (GeneID: 945365) cloned into EcoRI and HindII sites	Hobbs et al., 2012	N/A
pBAD24-*acrZ* P16A	This study	N/A
pBAD24-*acrZ* P16A V17P	This study	N/A
pBAD24-*acrZ* P16A V18P	This study	N/A
pBAD24-*acrZ* P16A M19P	This study	N/A
pBAD24-*acrZ* P16A A20P	This study	N/A
pBAD24-*acrZ* M19P	This study	N/A

(*Continued on next page*)

Continued

REAGENT or RESOURCE	SOURCE	IDENTIFIER
pBAD24-*acrZ* A20P	This study	N/A
pBAD24-*acrZ* P16G	This study	N/A
pBAD24-*acrZ* V15G P16G	This study	N/A
pBAD24-*acrZ* P16G V17G	This study	N/A
pBAD24-*acrZ* S7A	This study	N/A
pBAD24-*acrZ* V15A	This study	N/A
pBAD24-*acrZ* M19A	This study	N/A
pBAD24-*acrZ* I22A	This study	N/A
pBAD24-*acrZ* L23A	This study	N/A
pBAD24-*acrZ* I26A	This study	N/A
pBAD24-*acrZ* L29A	This study	N/A
pBAD24-*acrZ* G30A	This study	N/A
pBAD24-*acrZ* E31A	This study	N/A
pBAD24-*acrZ* F33A	This study	N/A
pEB354-*acrB* from *Escherichia coli* MG1655 (GeneID: 945108) cloned into XbaI and XhoI sites to create T25-AcrB fusion	Hobbs et al., 2012	N/A
pUT18-*acrZ* from *Escherichia coli* MG1655 (GeneID: 945365) cloned into HindIII and EcoRI sites to create AcrZ-T18 fusion	Hobbs et al., 2012	N/A
pUT18-*acrZ* P16A	This study	N/A
pUT18-*acrZ* P16A V17P	This study	N/A
pUT18-*acrZ* P16A V18P	This study	N/A
pUT18-*acrZ* P16A M19P	This study	N/A
pUT18-*acrZ* P16A A20P	This study	N/A
pUT18-*acrZ* M19P	This study	N/A
pUT18-*acrZ* A20P	This study	N/A
pUT18-*acrZ* P16G	This study	N/A
pUT18-*acrZ* V15G P16G	This study	N/A
pUT18-*acrZ* P16G V17G	This study	N/A
pUT18-*acrZ* S7A	This study	N/A
pUT18-*acrZ* V15A	This study	N/A
pUT18-*acrZ* M19A	This study	N/A
pUT18-*acrZ* I22A	This study	N/A
pUT18-*acrZ* L23A	This study	N/A
pUT18-*acrZ* I26A	This study	N/A
pUT18-*acrZ* L29A	This study	N/A
pUT18-*acrZ* G30A	This study	N/A
pUT18-*acrZ* E31A	This study	N/A
pUT18-*acrZ* F33A	This study	N/A
Software and Algorithms		
GROMACS	Abraham et al., 2015	http://manual.gromacs.org/documentation/2020.1/download.html
ISOLDE	Croll, 2018	https://isolde.cimr.cam.ac.uk/
PHENIX	Liebschner et al., 2019	https://www.phenix-online.org/
RELION 3.0	Scheres, 2012; Zivanov et al., 2018	https://www3.mrc-lmb.cam.ac.uk/relion/index.php/Main_Page
Motioncor2	Zheng et al., 2017	https://emcore.ucsf.edu/ucsf-motioncor2
Gctf	Zhang, 2016	https://www.mrc-lmb.cam.ac.uk/kzhang/
Chimera	Pettersen et al., 2004	https://www.cgl.ucsf.edu/chimera/

(*Continued on next page*)

Continued

REAGENT or RESOURCE	SOURCE	IDENTIFIER
ResMap	Kucukelbir et al., 2014	https://sourceforge.net/projects/resmap-latest/
PyMOL	DeLano, 2002	https://pymol.org/2/
Coot	Emsley et al., 2010	http://www.ccp4.ac.uk/index.php
EMRinger	Barad et al., 2015	https://github.com/fraser-lab/EMRinger
MOLPROBITY	Chen et al., 2010	http://molprobity.biochem.duke.edu
CHARMM-GUI Membrane Builder	Qi et al., 2015	http://www.charmm-gui.org/?doc=input/membrane.bilayer
LINCS	Hess et al., 1997	N/A
VMD	Humphrey et al., 1996	https://www.ks.uiuc.edu/Development/Download/download.cgi?PackageName=VMD

LEAD CONTACT AND MATERIALS AVAILABILITY

Further information and requests for resources and reagents should be directed to and will be fulfilled by the Lead Contact, Ben F. Luisi, bfl20@cam.ac.uk

All unique reagents generated in this study are available from the Lead Contact without restriction.

EXPERIMENTAL MODEL AND SUBJECT DETAILS

Constructs of AcrB/DARPin and AcrBZ/DARPin complexes were previously described and grown in C43(DE3) acrab (Du et al., 2014; Eicher et al., 2012). Drug sensitivity assays used strains MG1655 and MG1655 Δ*acrZ*::*kan* described earlier (Hobbs et al. 2012), though the *kan* marker in Δ*acrZ*::*kan* was removed using pCP20. Strain MG1655 Δ*clsABC*::FRT-*kan*-FRT (cardiolipin-deficient) was kindly provided by Douglas B. Weibel (Oliver et al. 2014). To prepare the Δ*clsABC* single and Δ*acrZ* Δ*clsABC* double mutation strains also used for the drug sensitivity assays, Δ*clsABC*::FRT-*kan*-FRT was transduced into the MG1655 and MG1655 Δ*acrZ* backgrounds following a standard P1 transduction protocol for *E. coli* genome manipulation (Thomason et al., 2007). MG1655 Δ*acrZ* (GSO284) was the background strain for all assays of *acrZ* mutants (Hobbs et al., 2010). BTH101 was the background strain for the two-hybrid assays (Hobbs et al., 2012). *E. coli* shuffle-T7 cells were used to express human saposin A in LB medium containing carbenicillin (100 μg/ml).

METHOD DETAILS

Preparation of Saposin A

Human saposin A was expressed in *E. coli* shuffle-T7 cells. Colonies of shuffle-T7/pET15b-saposin A from a freshly transformed plate were used to inoculate 50 ml of LB medium containing carbenicillin (100 μg/ml) in a 250-ml baffled flask. The cells were grown in an orbital shaker at 30°C, 220 rpm overnight. 20 ml of the starter culture was used to inoculate 1000 ml LB medium (with carbenicillin) in a 2-l baffled flask at 30°C, 220 rpm, and cultures were induced at A_{600}=0.6 – 0.8 with 1 mM isopropyl 1-thio-b-D-galactopyranoside (IPTG). The temperature was dropped to 16°C and the cells were grown at 220-rpm overnight. Cells were harvested by centrifugation at 4,200 rpm (Beckman 4.2 rotor) for 25 min at 4°C. Cell pellets from a 2-l culture were resuspended in 50 ml AEX buffer (50 mM Tris-HCl, pH 7.4, 25 mM NaCl) with 1 tablet EDTA-free protease inhibitor cocktail tablets and 5U/ml DNase I. Cells were lysed using a homogenizer (Emulsiflex) at 15,000 psi and the cell debris was pelleted by centrifugation at 40,000 xg for 30 min at 4°C. The supernatant was collected in a glass bottle, and was heat-treated at 85°C in a water bath for 10 min with gentle shaking, followed by centrifugation at 40,000 xg for 30 mins at 4°C. The supernatant was loaded onto a HiTrap Q column (GE Healthcare) equilibrated with AEX buffer. The column was washed with 10 column volumes of AEX buffer and then saposin A was eluted over a 0 – 50% gradient of AEX elution buffer (20 mM Tris, pH 7.4, 1 M NaCl). The fractions containing saposin A were pooled, and the protein was concentrated to 0.5 ml using 3 kDa Vivaspin concentrator. A final gel filtration step was performed using a Superdex 200 column equilibrated with GF Buffer-1 (20 mM HEPES pH 7.0, 150 mM NaCl). The peak fractions were concentrated, flash frozen in liquid nitrogen and stored at -80°C.

Protein Expression, Purification and Nanodisc Reconstitution

AcrB/DARPin and AcrBZ/DARPin complex purification followed the procedure described earlier (Du et al., 2014). The expression construct pET21a-acrB$_{\Delta His}$ has the C-terminal histidines of AcrB substituted to prevent nonspecific association with the metal affinity matrix. pET21a-acrB$_{\Delta His}$ and pRSFDuet-1-acrZ$_{His5}$ were transformed into *E. coli* strain C43(DE3) Δ*acrAB* and cells were grown in

2xYT medium with 100 mg/ml carbenicillin and 50 mg/ml kanamycin at 37°C until the culture reached an absorbance, at 600 nm, of 0.5–0.6 and was then induced by the addition of 0.5mM isopropyl 1-thio-b-D-galactopyranoside (IPTG) at 18°C overnight. Cell pellets were resuspended in lysis buffer (400 mM NaCl, 20 mM Tris-HCl, pH 8.0) with 1 tablet per 50 ml EDTAfree protease inhibitor cocktail tablets, 5 U/ml DNase I and 5 mg/ml lysozyme, and the mixture was stirred at 4°C for 1 h the passed 8 times through a homogenizer (EmulsiFlex) at 15,000 psi. Cell debris was removed by centrifugation at 9,000 ×g for 30 min. Cellular membrane was pelleted by ultracentrifugation at 125,755g for 3 h, and pellets resuspended in lysis buffer with protease inhibitors and were solubilized by adding 1.5% DDM and stirring at 4°C for 3 h. Debris was pelleted by ultracentrifugation at 125,755 ×g for 30 min. Imidazole was added to the membrane solution to a final concentration of 10mM. Histidine-tagged AcrBZ complex was purified by nickel affinity chromatography using a HiTrap chelating column (GE Healthcare Life Sciences) equilibrated with GF buffer 1 (400 mM sodium chloride, 20 mM Tris-HCl, pH 8.0, 0.05% DDM) containing 20 mM imidazole.

The column was washed with 50 mM and 75 mM imidazole added to GF buffer 1, respectively. Purified AcrBZ complex was eluted with 500 mM imidazole in GF buffer 1, concentrated and loaded onto a Superose 6 column equilibrated with GF buffer 1. Fractions containing purified AcrBZ complex were pooled and concentrated to 15–20 mg/ml using a Vivaspin concentrator (100 kDa MWCO) and dialysed overnight against sample buffer (10 mM HEPES pH 7.5, 50 mM NaCl, 0.03% DDM) using a 100 kDa MWCO dialysis membrane to decrease the detergent concentration. Purified DARPin and AcrBZ complex were mixed at a molar ratio of 1:2 (AcrBZ monomer:DARPin monomer). The mixture was diluted with GF buffer 2 (20 mM Tris-HCl, pH 7.5, 150 mM NaCl, 0.03% DDM) to a concentration of 2–3 mg/ml, incubated at 4°C overnight, then concentrated to 0.5 ml using a Vivaspin concentrator (MWCO 100 kDa) and loaded onto a Superose 6 column equilibrated with GF buffer 2. Fractions containing purified AcrBZ–DARPin complex were pooled and concentrated to 15–20 mg/ml using a Vivaspin concentrator (MWCO 100 kDa) and dialysed overnight against sample buffer using a 100 kDa MWCO dialysis membrane; the final concentration was 10–15 mg/ml.The purified, concentrated proteins were frozen in liquid nitrogen and stored at -80°C.

Nanodiscs were reconstituted using a modification of a procedure described earlier (Frauenfeld et al., 2016). Efficient nanodisc formation requires a step at pH 4, but AcrB and AcrBZ were unstable under this acidic condition. Thus, in the first step of the reconstitution procedures, nanodiscs were prepared with *E. coli* lipids at acidic pH and then brought to pH 7. In the second step, purified AcrB and AcrBZ were reconstituted into the pre-formed nanodiscs at the neutral pH.

For lipid stock preparation, 20 mg of *E. coli* total lipid extract (Avanti) was dissolved in 0.5 ml of chloroform. For lipid stock with extra cardiolipin, 20 mg of *E. coli* total lipid extract and 1 mg of cardiolipin were mixed and dissolved in 0.5 ml of chloroform. The lipid solutions in glass vials were evaporated in a vacuum desiccator, then resuspended in 1 ml of 50 mM HEPES pH 7.5, 150 mM NaCl and sonicated for 30 min. The lipid stocks were stored at -20°C. For the nanodisc reconstitution, 28.2 μl of saposin A (4.484 mM) was mixed with 50.8 μl of *E. coli* lipid stock (25 mM; for cardiolipin-enriched nanodiscs, 5% v/v purified cardiolipin (Avanti) was added to the lipid stock used for nanodisc formation), and then sodium acetate (50 mM pH 4.8) was added to the mixture to a final volume of 500 μl and incubated at 37°C for 10 min. 1 ml of GF buffer-2 (20 mM Tris-Cl pH 7.5, 150 mM NaCl) was added to the mixture, and the buffer was exchanged to GF Buffer-2 using a HiTrap Desalting 5 ml column. 2 ml of the eluate was mixed with AcrB/DARPin or AcrBZ/DARPin. The molar ratio of AcrB/DARPin or AcrBZ/DARPin:saposin A:lipid is about 1:10:100 in the reconstitution. The mixture was incubated at 4°C for half an h and dialyzed against 1000 ml of GF Buffer-2 overnight. The sample was dialyzed again against 1000 ml of GF Buffer-2 for further 3 h. The buffer exchanged sample was concentrated to 500 μl and purified by gel filtration chromatography using a Superdex 200 column equilibrated with GF Buffer-2. The peak fractions containing the protein in nanodiscs were concentrated to 2 mg/ml. Minocycline was added to AcrB/nanodisc or AcrB/nanodisc-cardiolipin to a final concentration of 2 mM; chloramphenicol was added to AcrBZ/nanodisc or AcrBZ/nanodisc-cardiolipin to 1 mM. The mixtures were incubated for 1 h at 4°C, then flash frozen in liquid nitrogen and stored at -80°C.

Electron Cryo-Microscopy

For the structure determination of saposin A discs with AcrB and AcrBZ as well as both complexes with supplementary cardiolipin, a 4.0 μl aliquot at 2 mg/ml was applied onto holey carbon film supported by a 300-mesh R1.2/1.3 Quantifoil gold grid (Quantifoil) that had been previously glow discharged. The grid was blotted for 2.5 – 3.0 seconds and rapidly frozen in liquid ethane using a Vitrobot IV (FEI) at 4°C and 100% humidity. The grids were stored in liquid nitrogen before imaging. Zero-energy-loss images of frozen-hydrated AcrB disc particles were recorded automatically on an FEI Titan Krios electron microscope at 300 kV, using a slit width of 20 eV on a GIF Quantum energy filter and a Gatan K2-Summit direct electron detector (Gatan) in counting mode. Images of frozen-hydrated AcrB/nanodisc-cardiolipin, AcrBZ/nanodisc and AcrBZ/nanodisc-cardiolipin particles were acquired automatically on the FEI Titan Krios electron microscope at 300 kV using a Falcon III direct electron detector camera (FEI) in counting mode.

Image Processing and 3D Reconstruction

The software MotionCor2 (Zheng et al., 2017) was used for whole-frame motion correction and dose weighting, Gctf (Zhang, 2016) for estimation of the contrast transfer function parameters, and RELION-3.0 (Scheres, 2012; Zivanov et al., 2018) package for all other image processing steps. A particle subset was manually selected to calculate reference-free 2D class averages, which was then used as templates for automated particle picking of the entire data set. The templates were low-pass filtered to 20 Å to limit model bias. Then several runs of 2D classifications were used to remove the heterogeneous particles, as well as the false positive particles from the auto-picking. A reference map was generated from crystal structure of AcrB using the program pdb2mrc in the EMAN package (Ludtke et al., 1999), and was low pass filtered to 60 Å resolution and used as a starting point for the 3D classification. We selected

good particles for further analysis based on the quality and high resolution in the 2D and 3D classifications. The 3D auto-refinement resulted in near-atomic resolution maps. After per-particle motion correction and radiation-damage weighting by Bayesian polishing in RELION (Zivanov et al., 2018), the polished particles were subjected to 2D and 3D classifications, and 3D auto-refinement again. A soft mask in RELION post-processing was applied before computing the FSCs. The overall resolution of the maps was estimated by the gold-standard FSC criterion with 0.143 cut-off (Rosenthal and Henderson, 2003). Local resolution variations were estimated with ResMap (Kucukelbir et al., 2014). The data collection and processing parameters for all four specimens are summarized in Table S1.

Model Docking and Refinement

Automated structural refinement using Rosetta followed the procedure described in (Wang et al., 2017b). The models were refined with PHENIX (Adams et al., 2010; Liebschner et al., 2019) and ISOLDE (Croll, 2018), and the structures were further modelled and visualized using PyMOL (DeLano, 2002), Chimera (Pettersen et al., 2004) and Coot (Emsley et al., 2010). The crystal structure of human saposin A in the open state was docked into the maps. Density was also apparent for lipids, and the corresponding hydrocarbon portion was modelled into the density where there was good correspondence. Although the AcrB/nanodisc specimens were prepared in the presence of minocycline, density for the compound was not apparent in the refined cryo-EM maps inside the periplasmic domain. However, weak density that is consistent with minocycline is present near F556 (subunit A). A more defined density was found in the AcrBZ/nanodisc-cardiolipin EM map and subsequently assigned to chloramphenicol, which was added to the sample prior to freezing. The quality of the stereochemistry was evaluated with EMRinger (Barad et al., 2015) and MOLPROBITY (Chen et al., 2010).

Molecular Dynamics Simulations

For the atomistic simulations, the proteins were parameterized using CHARMM36 force field (Huang and MacKerell, 2013), whilst the parameters for the antibiotic chloramphenicol were obtained using CHARMM-GUI ligand modeler (Kim et al., 2017). The AcrZ P16A M19P mutant was generated using PyMOL (DeLano, 2002). A small patch of 15 X 15 nm model membrane was constructed using CHARMM-GUI membrane builder (Jo et al., 2009) to mimic the lipid composition of *Escherichia coli* K12 inner membrane (Aibara et al., 1972; Lugtenberg and Peters, 1976; Yokota et al., 1980) (75% 1-palmitoyl 2-cis-vaccenic phosphatidylethanolamine (PVPE), 20% 1-palmitoyl 2-cis-vaccenic phosphatidylglycerol (PVPG) and 5% 1-palmitoyl 2-cis-vaccenic 3-palmitoyl 4-cis-vaccenic diphosphatidylglycerol (cardiolipin)). Protein insertion into the membrane was performed using the *g_membed* protocol in GROMACS (Wolf et al., 2010). This system was then solvated with the SPC water molecules (Lins and Hünenberger, 2005) and neutralised with 0.15 M NaCl. A short 1 ns equilibration simulation was performed whereby the heavy atoms of the protein were positionally restrained using a force constant of 1000 kJ mol^{-1}. The temperature was kept at 310 K using the Nose-Hoover thermostat with a time constant of 1.0 ps (Hoover, 1985; Nosé, 1984). The pressure was held at 1 atm using a semi-isotropic coupling to the Parrinello-Rahman barostat with a time constant of 1.0 ps (Parrinello and Rahman, 1981). The electrostatic interactions were calculated using the smooth particle mesh Ewald method with a real-space cut off value of 1.2 nm (Essmann et al., 1995). The twin range cut-off method with a cut-off value of 1.2 nm was used to calculate the Van der Waals interactions, whereby the forces were switched to zero between 1.0 to 1.2 nm. The LINCS algorithm was utilised to constrain all covalent bonds to their equilibrium value, allowing for a 2 fs time step (Hess et al., 1997). After this equilibration simulation, the position restrains on the protein were removed and two independent production runs, each for 500 ns, were conducted with different starting velocities. Steered molecular dynamic simulations were performed whereby a harmonic spring with a force constant of 100 kJ mol^{-1} nm^{-2} was attached to the center of mass of chloramphenicol and pulled at a constant velocity of 0.1 nm ns^{-1} towards a reference residue (F136) found in the deep binding pocket of the L protomer. Three independent steered molecular dynamic simulations were performed for each of AcrB and AcrBZ starting with different velocities.

For the coarse-grained simulations, the proteins were converted to coarse-grained representation using the *martinize.py* script using the MARTINI 2.2 force field (Monticelli et al., 2008) with ElNeDyn to retain the secondary and tertiary structures (Periole et al., 2009). A patch of 30 x 30 nm membrane model of the same lipid composition as the atomistic simulation was constructed using CHARMM-GUI Martini Maker Bilayer Builder (Qi et al., 2015). The protein was placed in the middle of the membrane and overlapping lipids were removed. To understand the interactions between the protein and charged lipids, we reorganized the position of POPG and cardiolipin such that at the beginning of the simulation there were none of these lipids within 30 Å of the protein. The simulation box was solvated with the standard MARTINI water molecules and neutralised with 0.15 M NaCl. Energy minimisation was then performed using the steepest descent method. The system was subsequently equilibrated for 10 ns with positional restrains applied on the protein. The temperature was maintained at 310 K using a velocity-rescaling thermostat (Bussi et al., 2007), with a relaxation time of 1 ps, whereas the pressure was maintained at 1 bar by a semi-isotropic coupling with the Berendsen barostat (Berendsen et al., 1984) and a time constant of 5 ps. The cut-off for the nonbonded interactions was set at a distance of 1.2 nm, while the Lennard-Jones and Coulomb potentials were shifted from 0.9 and 0.0 to the cut-off distance, respectively. The LINCS algorithm was used to constrain all covalent bonds to their equilibrium values (Hess et al., 1997) and the time step was slightly increased from 2, 5, 8 to 10 fs to allow the system to be well equilibrated. After the equilibration simulations, protein position restraints were removed and three independent production runs with different starting velocities were performed for 5 μs using the same parameters, except a Parrinello-Rahman barostat with a time constant of 12 ps was used to control the pressure (Parrinello and Rahman, 1981).

All simulations were performed using the GROMACS 5 package (Abraham et al., 2015) and visualised in VMD (Humphrey et al., 1996). The bending angle of AcrZ was determined using the Bendix plug-in within VMD (Dahl et al., 2012). Protein-lipid contact

analysis was performed using *gmx select*. The partial mass density landscapes was generated with a modified version of the *g_density* tool (Castillo et al., 2013).

Bacterial Two-Hybrid Assays

Single colonies of a *E. coli* strain adenylate cyclase deletion strain (BTH101) freshly transformed with plasmids bearing *acrZ* fused to the T18 fragment of adenylate cyclase on the C-terminus and *acrB* fused to the T25 fragment of adenylate cyclase on the N-terminus were grown in 1 ml of LB medium supplemented with 30 μg/ml kanamycin and 50 μg/ml carbenicillin at 37°C with shaking at 250 rpm overnight. The cultures were diluted 1:100 into 3 ml of the same medium also containing 1 mM IPTG inducer and were similarly grown to $OD_{600} \sim 1$. An aliquot of sample was used for β-galactosidase assays as previously described (Miller, 1992) with some modifications. The OD_{600} was recorded and a 100 μl aliquot was added to a 1.5 ml microfuge tube containing 700 μl Z buffer (60 mM Na_2HPO_4, 40 mM NaH_2PO_4, 10 mM KCl, 1 mM $MgSO_4$, 2.7 μl/ml of β-mercaptoethanol, 1.5 μl/ml of 0.1% SDS) and 30 μl chloroform. The samples were vortexed and incubated for 15 min at 28°C, whereupon 100 μl of 8 mg/ml ortho-nitrophenyl-β-galactoside (ONPG) in Z buffer was added to the sample and vortexed and returned to 28°C. Once a yellow colour developed (~10 min), 0.5 ml of 1 M Na_2CO_3 was added and the sample was vortexed. The start times (addition of ONPG) and the stop times (addition of Na_2CO_3) were recorded. The sample was centrifuged to remove pellet debris and the A_{420} of the supernatant was recorded. Miller Units were calculated as follows: $(1000 \times A_{420}) \div (t \times v \times OD_{600})$, where t is min and v is ml.

Gradient Plate Assay

Gradient plates were prepared as previously described (Bryson and Szybalski, 1952) with slight modifications. In brief, a 100 mm x 100 mm x 1.5 mm square petri dish (Thomas Scientific) was propped up on a 1 cm ledge to create a slant. 40 ml of LB with 1.5% agar and either 10 μg/ml chloramphenicol or 300 μg/ml erythromycin was poured and allowed to harden. The plate was then laid flat and 40 ml of LB with 1.5% agar was overlaid. For strains carrying plasmids, both top and bottom agar were also supplemented with 50 μg/ml carbenicillin and 0.02% arabinose. Colonies for each strain isolated from sterile LB-Agar plates were grown overnight in LB (with carbenicillin for plasmid-containing strains) at 37°C with shaking at 250 rpm. The overnight cultures were diluted 1:100 and grown in the same media at 37°C with shaking at 250 rpm to exponential phase (OD_{600}~0.4). Aliquots (15 μl) were dripped down the plate starting from the edge with the lowest antibiotic concentration. The drips were allowed to dry, and the plates were incubated at 37°C for 16 h and imaged using the fluorescein setting on a ChemiDoc MP Imaging machine (Biorad). Each gradient plate assayed cultures generated from separate single colonies. Reproducible results were observed for 10 gradient plate assays conducted over three separate days. A representative image was selected for publication.

Cell Growth for Drug Sensitivity Assay

Colonies from *E. coli* MG1655 (wild type parent strain), MG1655 Δ*acrZ*, MG1655 Δ*clsABC*::FRT-*kan*-FRT (cardiolipin-deficient) and MG1655 Δ*clsABC*::FRT-*kan*-FRT Δ*acrZ* (cardiolipin-deficient and Δ*acrZ*) isolated from sterile LB-Agar plates were grown in LB for about 3 h at 37°C and then used to inoculate fresh medium in a 96-well plate. The cells were diluted to OD_{600nm} ~0.06 in fresh LB medium in the wells of a 96-well plate to which antibiotics were added as indicated in Figure 5. Growth was followed over time at OD_{660} at 37°C in a CLARIOstar microplate reader (BMG LABTECH). For the determination of the relative growth rates of the cultures in each of the wells, the exponential phase of the growth curve (as a mean of n = 3 for each culture type) was determined from the linear increase in a $\log_{10}(OD_{660nm})$ versus time plot. The slope of this section was determined by simple linear regression. Heteroscedasticity-consistent standard errors of the corresponding slope coefficient were calculated. The quality of the fit was significant in all cases ($P < 0.05$). Next, the relative growth rate was determined as the ratio of the growth rate in the presence of drug over the maximum growth rate in the absence of drug.

QUANTIFICATION AND STATISTICAL ANALYSIS

Statistical analyses of data are described in the legends for Figures 4E, 4F, and 5A.

DATA AND CODE AVAILABILITY

The cryoEM maps and models are available in the PDB and EMDB databases with codes, respectively of 6SGS and 10183 for the AcrBZ/saposin A disc with chloramphenicol; 6SGU and 10185 for AcrB/saposin A disc with minocycline; 6SGR and 10182 for AcrBZ/saposin A disc with 5% cardiolipin and chloramphenicol; and 6SGT and 10184 for AcrB/saposin A disc with 5% cardiolipin and minocycline.

Structure, Volume *28*

Supplemental Information

Interactions of a Bacterial RND Transporter with a Transmembrane Small Protein in a Lipid Environment

Dijun Du, Arthur Neuberger, Mona Wu Orr, Catherine E. Newman, Pin-Chia Hsu, Firdaus Samsudin, Andrzej Szewczak-Harris, Leana M. Ramos, Mekdes Debela, Syma Khalid, Gisela Storz, and Ben F. Luisi

Supplementary Information

Interactions of a bacterial RND transporter with a transmembrane small protein in a lipid environment

Dijun Du, Arthur Neuberger, Mona Wu Orr, Catherine E. Newman, Pin-Chia Hsu, Firdaus Samsudin, Andrzej Szewczak-Harris, Leana M. Ramos, Mekdes Debela, Syma Khalid, Gisela Storz, Ben F. Luisi

correspondence bfl20@cam.ac.uk, S.Khalid@soton.ac.uk, dudj@shanghaitech.edu.cn, or storzg@mail.nih.gov

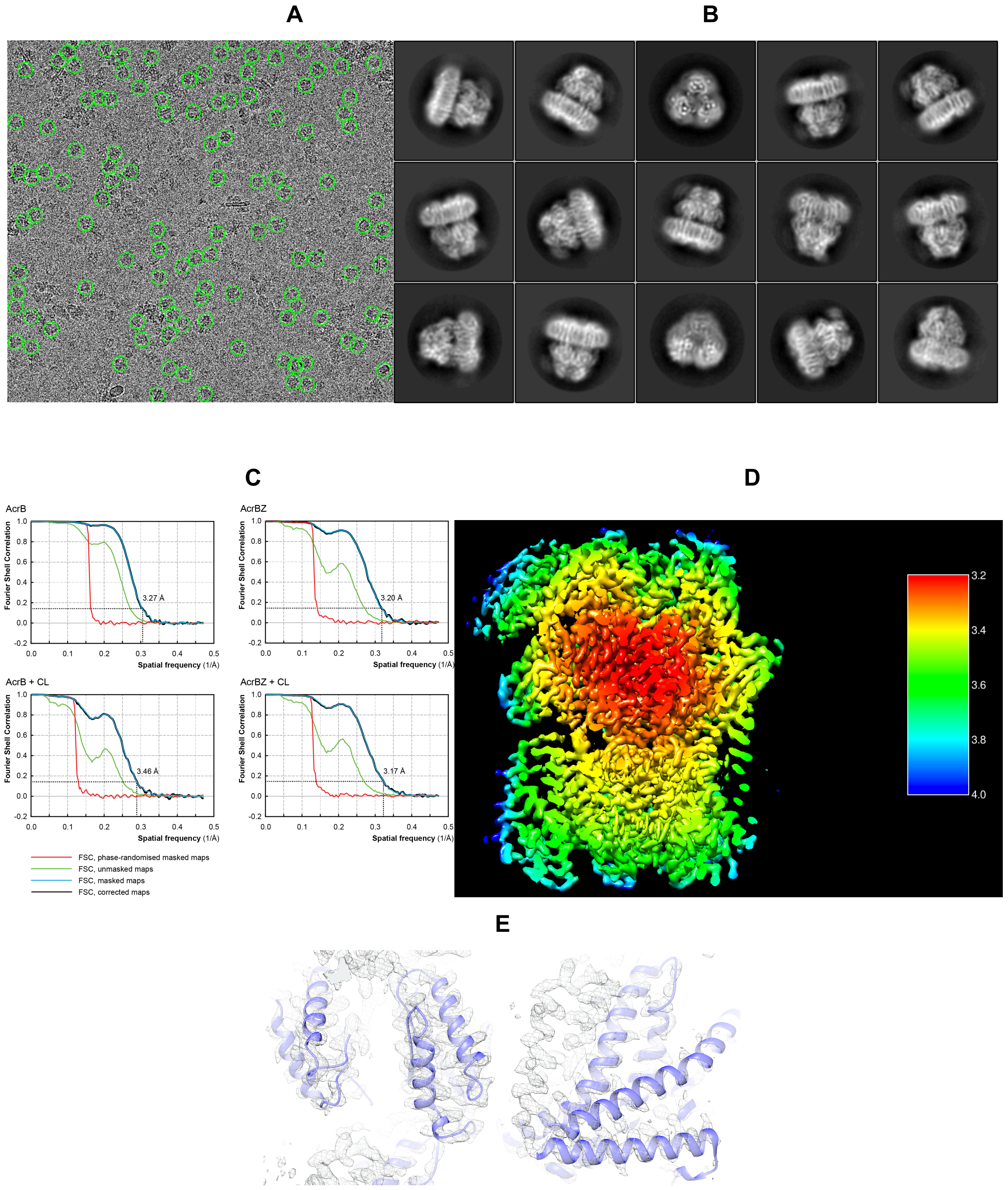

Figure S1. Related to Figure 1. Cryo-EM of AcrBZ in saposin A disc. (**A**) Representative cryo-EM micrograph image of AcrBZ in saposin A disc. The image has been corrected for drift. (**B**) Typical two-dimensional class averages of the particles. (**C**) Gold-standard Fourier Shell Correlation indicating the resolution of the density maps. Shown are FSC plots generated between reconstructions from random halves of the data. (**D**) Local resolution estimation by ResMap of AcrBZ. (**E**) Details of

structure-map overlay reveals quality of fit suitable to validate observed structural differences.

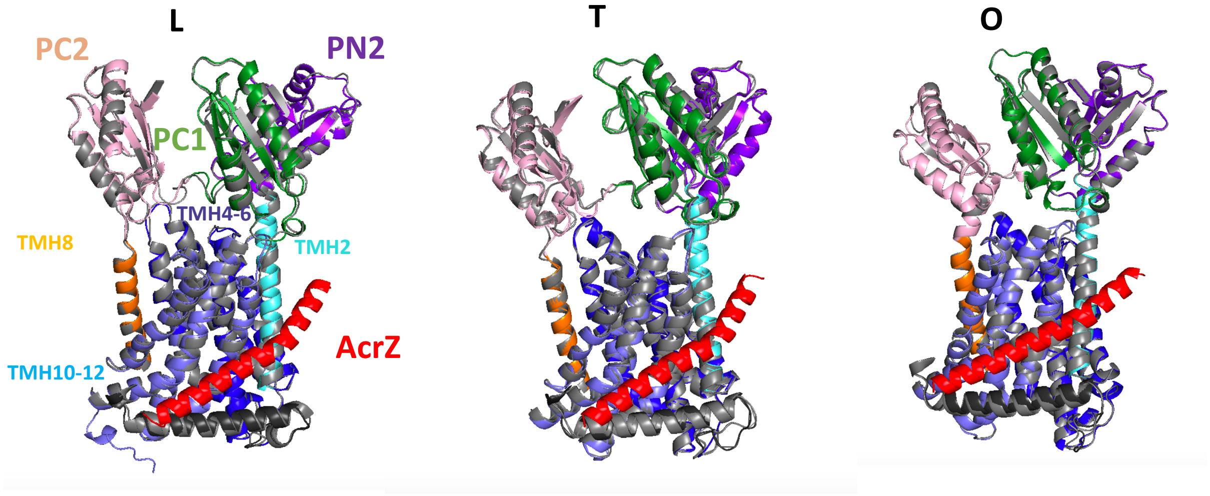

Figure S2. Related to Figure 3. Structural changes associated with AcrZ binding. Overlay of AcrB (grey; partially transparent) and AcrBZ (colored) protomers in L, T, O, conformation from cryo-EM derived structures of protein(s) reconstituted in *E. coli* lipids inside a saposin A-disc. To facilitate visualisation, only part of the protein is displayed for which changes were greatest: PC1/2, PN2, I2, TMH 2, TMH 8, TMH 10-12, and TMH 4-6 (i.e. reference frame). Color Code: PC2, pink; PN2, purple; PC1, dark green; TMH 8, orange; TMH 4-6, navy blue; TMH 2, cyan; TMH 10-12, blue; I2, grey.

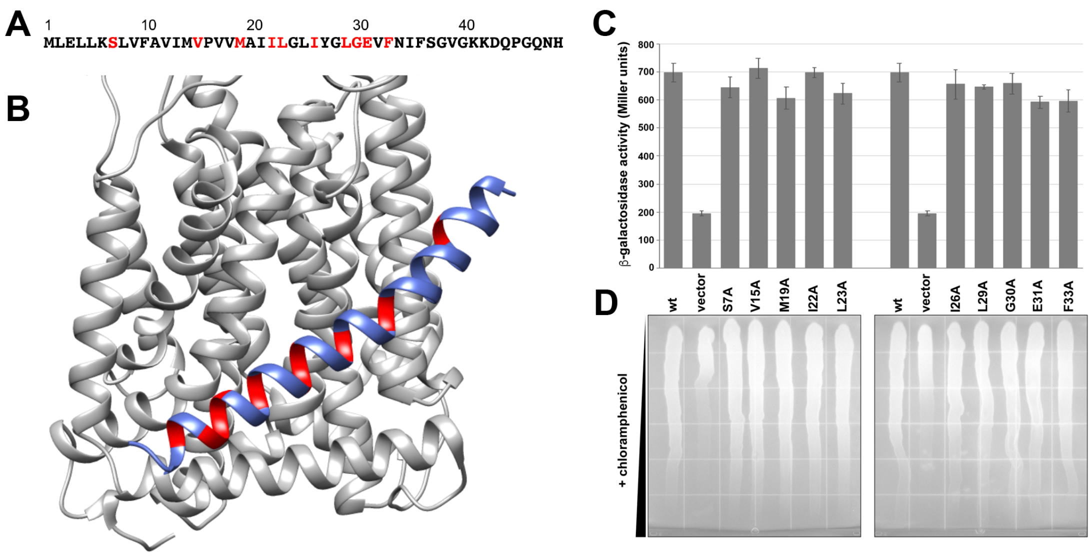

Figure S3. Related to Figure 4. Mutation of AcrZ interfacial residues. (**A**) The sequence of AcrZ. Interfacial residues predicted by previous structure and mutated are highlighted in red. (**B**) Structure of AcrB (grey) and AcrZ (blue) with interfacial residues indicated in red. (**C**) Split adenylate cyclase two-hybrid assays of the interaction between plasmid-encoded T25-AcrB and the empty vector, wild type AcrZ-T18 or the indicated mutant. T25-AcrB and the AcrZ-T18 indicated were co-expressed in an adenylate cyclase deficient strain and grown to OD_{600} ~ 1 when cells were harvested for β-galactosidase activity assay. Shown are the average and standard deviation of three experiments. The first and second wt and vector samples are the same. (**D**) Exponentially-growing cultures of the *E. coli* Δ*acrZ* strains carrying the pBAD24 empty vector, wild type AcrZ or the indicated AcrZ mutant were applied across chloramphenicol gradient plates to visualize differences in antibiotic sensitivity. The plates were incubated overnight at 37°C and photographed. Shown here is a representative image of an experiment carried out in triplicate.

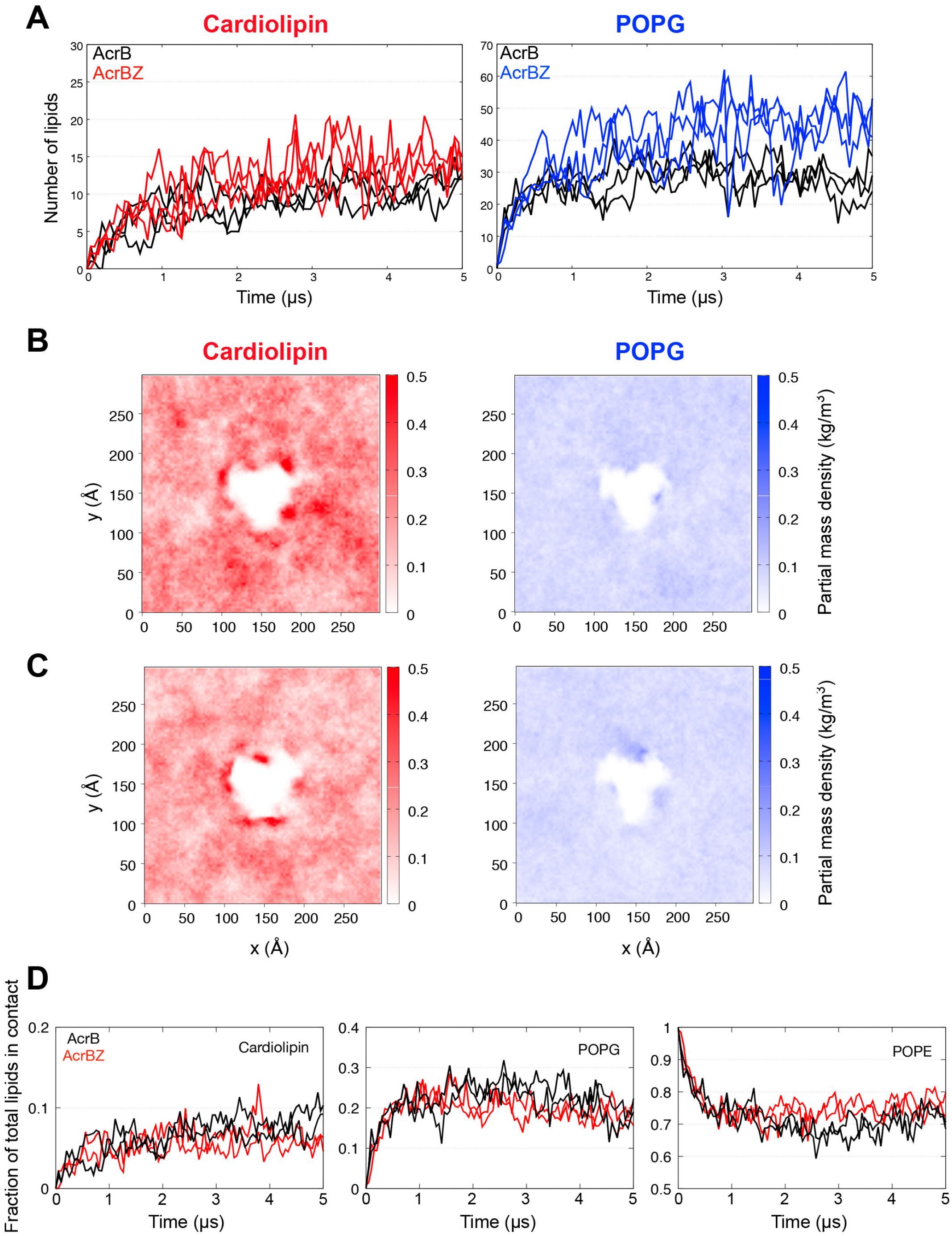

Figure S4. Related to Figure 5. Cardiolipin and POPG enrichment around AcrB and AcrBZ. (**A**) The number of cardiolipin (left) and POPG (right) found within 6 Å of AcrB (black) and AcrBZ (red or blue) throughout 5 µs of coarse-grained simulations. Data shown from three independent repeats. (**B**) Partial mass density of cardiolipin (red) and POPG (blue) and around AcrB (white space in the middle of the graph). This is normalized to the number of cardiolipin and POPG in the membrane. (**C**) Same

analysis performed on AcrBZ. (**D**) The number of each lipid species divided by the total number of lipids in contact with AcrB and AcrBZ during the simulation.

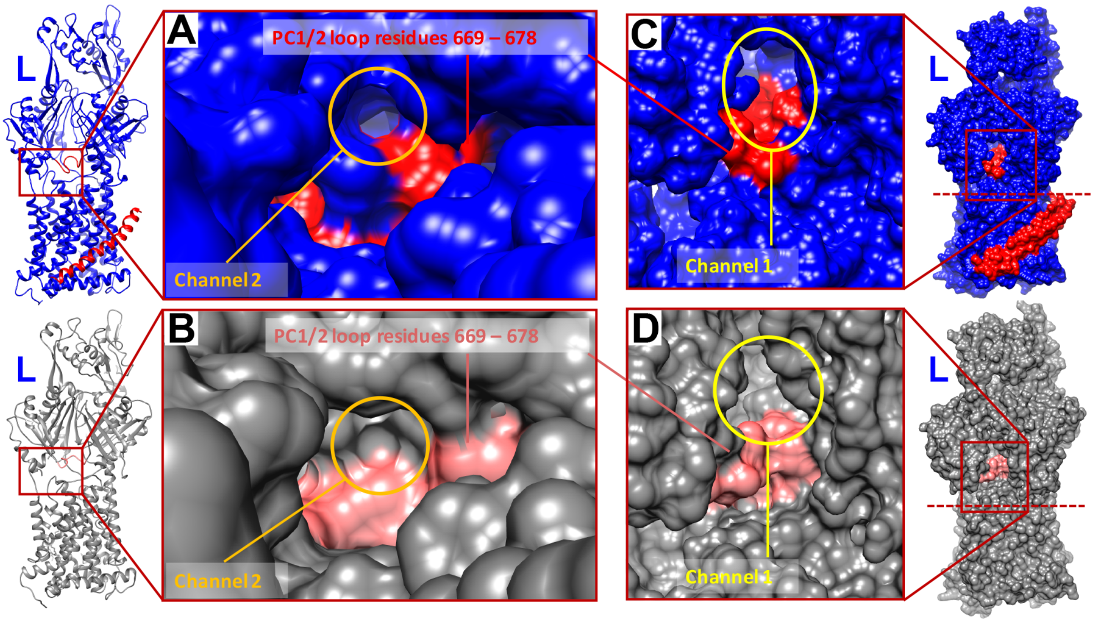

Figure S5. Related to Figure 6. Structural comparison of substrate entry site between saposin A disc reconstituted AcrB and AcrBZ with cardiolipin enrichment. L protomers of cryo-EM derived AcrB (in grey; partially transparent) reconstituted in *E. coli* lipids inside a saposin A disc and AcrBZ (blue) reconstituted in *E. coli* lipids enriched with cardiolipin inside a saposin A disc. Channel 2 entry is restricted by a loop region of PC1/2 (red) for substrate entry from the outer leaflet of the inner membrane. In the surface view, the entry site is open in case of the AcrBZ complex with 5% cardiolipin addition (**A**) and closed in AcrB without cardiolipin enrichment (**B**). The channel 1 entry site, protruding from the periplasm, is slightly lowered in case of AcrBZ (**C**), for easier access from above the membrane outer surface area, and elevated in case of AcrB (**D**). The dashed lines indicate the approximate level of the outer leaflet boundary with the periplasm (at which substrates presumably enter the complex).

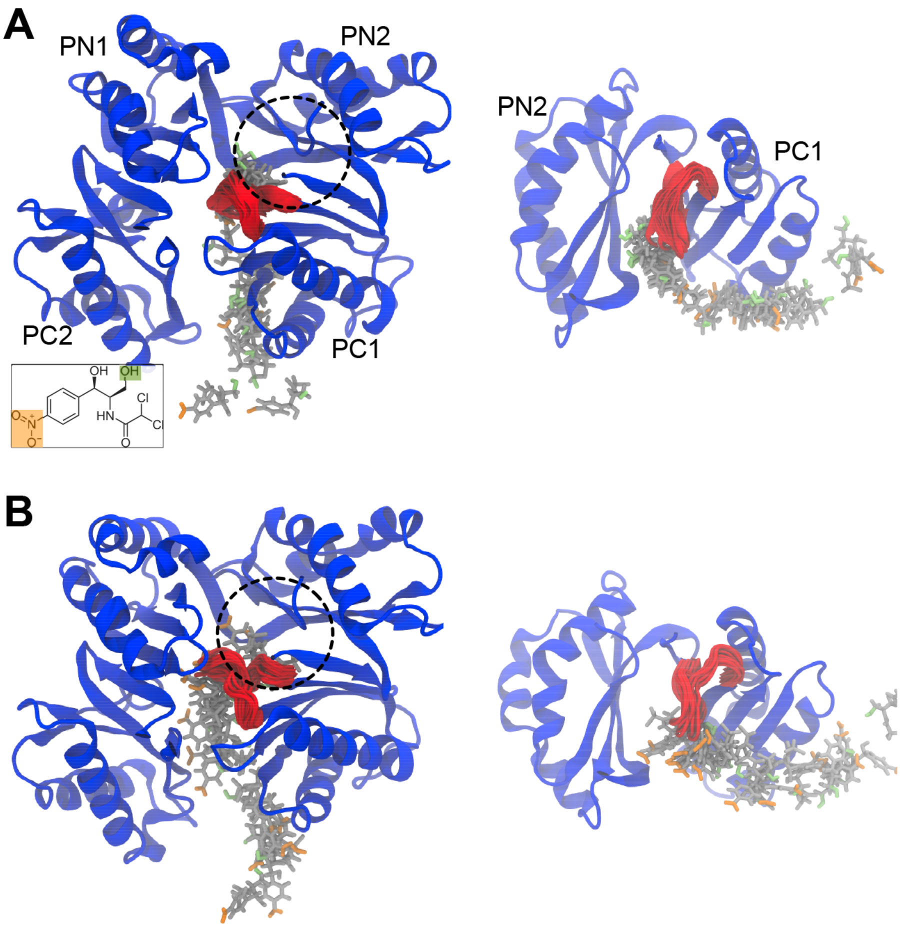

Figure S6. Related to Figure 6. Translocation of chloramphenicol into the binding site of AcrBZ and AcrB unaffected by the switch loop conformation. (**A**) A steered molecular dynamic simulation was performed whereby a chloramphenicol molecule was pulled from the periplasmic space into the deep binding pocket of the L protomer of AcrBZ. Views from the top (left) and side (right) of the protein are shown. Snapshots of chloramphenicol (nitrite group is orange, hydroxyl is green, and the rest is grey as shown in inset) and the switch loop (red) were taken every 1 ns of the simulation. The approximate position of the deep binding pocket is indicated by the black dotted circle. (**B**) A steered molecular dynamic simulation performed for AcrB showing a similar entry pathway of the drug into the protein. These simulations indicate that the switch loop is flexible and is able to allow the passage of chloramphenicol for both AcrBZ and AcrB.

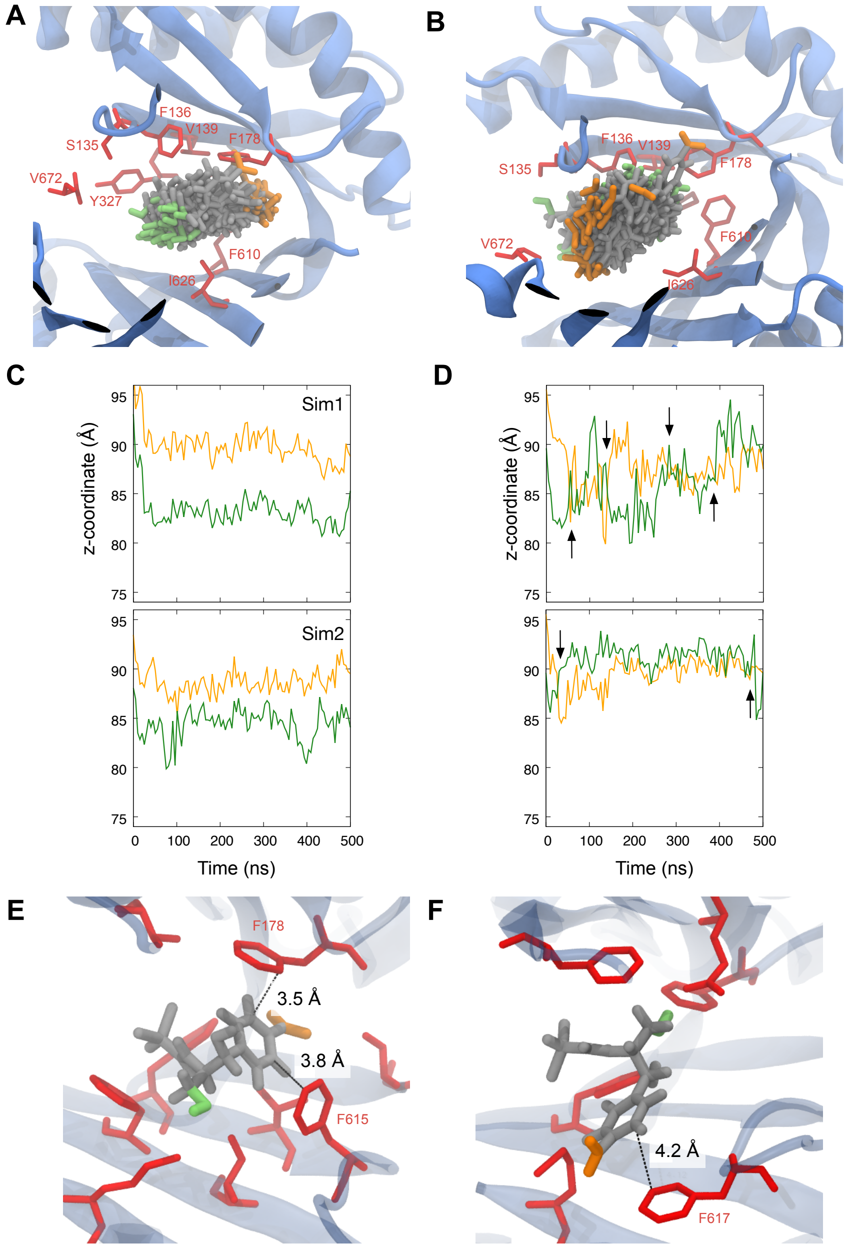

Figure S7. Related to Figure 6. Chloramphenicol binds more stably to AcrBZ with 5% cardiolipin. (**A**) and (**B**) Snapshots of chloramphenicol (same color scheme as Figure S6 for the antibiotic and Figure 6C,D for protein) bound to the binding site of the T protomer of AcrBZ and AcrB, respectively, taken every 10 ns from a 500 ns simulation. Residues highlighted in Figure 6C,D are shown in red. (**C**) and (**D**) The coordinate along the z-axis (perpendicular to the plane of the membrane) of the nitrite and hydroxyl moieties during two independent simulations (Sim1 and Sim2) with

AcrBZ and AcrB, respectively. Arrows in (**D**) indicate the time points at which the drug molecule flipped its orientation within the binding site. (**E**) and (**F**) The most frequently sampled conformation of the drug molecule from these simulations. The binding of chloramphenicol to AcrBZ was potentially stabilized by a pi-stacking interaction between its nitrobenzene ring and either F178 and F615. This interaction was absent in the AcrB simulations and the closest aromatic residue was F617, which was more than 4 Å away.

Table S1. Cryo-EM data collection and refinement statistics for AcrBZ and AcrB structures, Related to Figure 1.

**Structure**	AcrBZ saposin A disc Chloramphenicol	AcrB saposin A disc Minocycline	AcrBZ saposin A disc + 5% cardiolipin Chloramphenicol	AcrB saposin A disc + 5% cardiolipin Minocycline
PDB code	6SGS	6SGU	6SGR	6SGT
EMDB code	10183	10185	10182	10184
**Data collection**				
Microscope	FEI Titan Krios	FEI Titan Krios	FEI Titan Krios	FEI Titan Krios
Voltage (kV)	300	300	300	300
Detector	Falcon III	K2	Falcon III	Falcon III
Mode	Counting	Counting	Counting	Counting
Nominal magnification	75,000 ×	130,000 ×	75,000 ×	75,000 ×
Pixel size (Å)	1.09	1.06	1.09	1.06
Electron dose, per frame (e^-/Å^2)	0.350	1.335	0.586	0.919
Electron dose, total (e^-/Å^2)	26.25	53.4	46.9	44.1
Defocus range (μm), step (μm)	–1.3 to –3.4, 0.3	–1.3 to –3.4, 0.3	–1.3 to –3.4, 0.3	–1.3 to –3.4, 0.3
Exposure (s)	60	20	60	70
Frames	75	40	80	48
Number of micrographs	1,356	2,156	2,720	580
**Reconstruction**				
Software	RELION-3.0	RELION-3.0	RELION-3.0	RELION-3.0
Number of particles used	94,507	414,786	95,775	77,593
Final resolution, $FSC_{0.143}$ (Å)	3.20	3.27	3.17	3.46
Map-sharpening B factor (Å^2)	–107.0	–101.2	–99.6	–109.1
**Model composition**				
Non-hydrogen atoms	54,156	52,169	54,150	52,180
Protein residues	3,528	3,407	3,528	3,408
**Refinement**				
Software	Phenix-1.15.2	Phenix-1.15.2	Phenix-1.15.2	Phenix-1.15.2
Correlation coefficient, masked	0.82	0.82	0.83	0.79
Correlation coefficient, box	0.65	0.71	0.69	0.65
**Validation (proteins)**				
MolProbity score	1.12	1.39	1.19	1.49
Clash score, all atoms	2.03	3.26	2.14	3.28
EMRinger score	3.39	1.65	3.52	2.47
**Ramachandran plot statistics**				
Favoured, overall (%)	97.21	96.03	96.78	95.38
Allowed, overall (%)	2.79	3.94	3.22	4.53
Outlier, overall (%)	0.00	0.03	0.00	0.09
**R.m.s. deviations**				
Bond length (Å)	0.003	0.004	0.003	0.003
Bond angle (°)	0.581	0.634	0.533	0.633

Table S3. Oligonucleotides. Related to Figure 4.

JK1 (Fwd QuikChange Lightning Primer *acrZ* P16G)	CTGGTATTCGCCGTAATCATGGTAgggGTCGTGATGGCCA
JK2 (rev QuikChange Lightning Primer *acrZ* P16G)	TGGCCATCACGACcccTACCATGATTACGGCGAATACCAG
JK3 (Fwd QuikChange Lightning Primer *acrZ* V15G P16G)	GGTATTCGCCGTAATCATgggtggTGTCGTGATGGCCATCATCC
JK4 (rev QuikChange Lightning Primer *acrZ* V15G P16G)	GGATGATGGCCATCACGACAccacccATGATTACGGCGAATACC
JK5 (Fwd QuikChange Lightning Primer *acrZ* P16G V17G)	CTGGTATTCGCCGTAATCATGGTAggggggGTGATGGCCATCATCCTGG
JK6 (Rev QuikChange Lightning Primer *acrZ* P16G V17G)	CCAGGATGATGGCCATCACccccccTACCATGATTACGGCGAATACCAG
lr11 (fwd Q5 mutagenesis primer *acrZ* P16A A20P)	CGTCGTGATGcctATCATCCTGG
lr12 (rev Q5 mutagenesis primer *acrZ* P16A A20P)	GCTACCATGATTACGGCG
lr19 (fwd Q5 mutagenesis primer *acrZ* P16A V17P)	CATGGTAGCCcctGTGATGGCCATC
lr20 (rev Q5 mutagenesis primer *acrZ* P16A V17P)	ATTACGGCGAATACCAGAC
lr21 (fwd Q5 mutagenesis primer *acrZ* P16A V18P)	GGTAGCCGTCcctATGGCCATCATC
lr22 (rev Q5 mutagenesis primer *acrZ* P16A V18P)	ATGATTACGGCGAATACC
lr23 (fwd Q5 mutagenesis primer *acrZ* P16A M19P)	AGCCGTCGTGcctGCCATCATCC
lr24 (rev Q5 mutagenesis primer *acrZ* P16A M19P)	ACCATGATTACGGCGAATAC
lr40 (fwd QuikChange Lightning primer *acrZ* M19P)	CATGGTACCTGTCGTGccgGCCATCATCCTGGGTC
lr41 (rev QuikChange Lightning primer *acrZ* M19P)	GACCCAGGATGATGGCcggCACGACAGGTACCATG
lr46 (fwd QuikChange Lightning primer *acrZ* I26A)	ATGGCCATCATCCTGGGTCTGgcgTACGGTCTTGGTGAAGTATTC
lr47 (rev QuikChange Lightning primer *acrZ* I26A)	GAATACTTCACCAAGACCGTAcgcCAGACCCAGGATGATGGCCAT
lr50 (fwd Q5 mutagenesis primer *acrZ* A20P)	TGTCGTGATGcctATCATCCTGG
lr51 (rev Q5 mutagenesis primer *acrZ* A20P)	GGTACCATGATTACGGCG
mo20 (fwd Q5 mutagenesis primer *acrZ* S7A)	GTTATTAAAAgcactaGTATTCGCCGTAATCATG
mo21 (rev Q5 mutagenesis primer *acrZ* S7A)	TCTAACATCAAGCTTGGC
mo26 (fwd Q5 mutagenesis primer *acrZ* V15A)	CGTAATCATGgcaccgGTCGTGATGG
mo27 (rev Q5 mutagenesis primer *acrZ* V15A)	GCGAATACCAGACTTTTTAATAAC
mo30 (fwd Q5 mutagenesis primer *acrZ* M19A)	ACCTGTCGTGgccGCCATCATCC
mo31 (rev Q5 mutagenesis primer *acrZ* M19A)	ACCATGATTACGGCGAATAC
mo32 (fwd Q5 mutagenesis primer *acrZ* I22A)	TCGTGATGGCgatcgcCCTGGGTCTG
mo33 (rev Q5 mutagenesis primer *acrZ* I22A)	CAGGTACCATGATTACGG

mo34 (fwd Q5 mutagenesis primer *acrZ* L23A)	GGCCATCATCgcaGGTCTG ATTTACG
mo35 (rev Q5 mutagenesis primer *acrZ* L23A)	ATCACGACAGGTACCATG
mo45 (fwd Q5 mutagenesis primer *acrZ* L29A)	CGGTCTTGGTgccGTATTCA ACATC
mo46 (rev Q5 mutagenesis primer *acrZ* L29A)	TAAATCAGACCCAGGATG
mo47 (fwd Q5 mutagenesis primer *acrZ* G30A)	gccGAAGTATTCAACATCTT TTCTGG
mo48 (rev Q5 mutagenesis primer *acrZ* G30A)	TAGCCCGTAAATCAGACCC AGG
mo49 (fwd Q5 mutagenesis primer *acrZ* E31A)	CGGTCTTGGTgccGTATTCA ACATC
mo50 (rev Q5 mutagenesis primer *acrZ* E31A)	TAAATCAGACCCAGGATG
mo51 (fwd Q5 mutagenesis primer *acrZ* F33A)	TTGGTGAAGTggccAACATC TTTTCTGG
mo52 (rev Q5 mutagenesis primer *acrZ* F33A)	GACCGTAAATCAGACCCA G
mo67 (fwd Q5 mutagenesis primer *acrZ* P16A)	AATCATGGTAgccGTCGTGA TGG
mo68 (rev Q5 mutagenesis primer *acrZ* P16A)	ACGGCGAATACCAGACTT
